# FET fusion oncoproteins interact with BRD4 and SWI/SNF chromatin remodelling complex subtypes in sarcoma

**DOI:** 10.1002/1878-0261.13195

**Published:** 2022-03-19

**Authors:** Malin Lindén, Christoffer Vannas, Tobias Österlund, Lisa Andersson, Ayman Osman, Mandy Escobar, Henrik Fagman, Anders Ståhlberg, Pierre Åman

**Affiliations:** ^1^ Department of Laboratory Medicine Sahlgrenska Academy Sahlgrenska Center for Cancer Research Institute of Biomedicine University of Gothenburg Sweden; ^2^ Wallenberg Centre for Molecular and Translational Medicine University of Gothenburg Sweden; ^3^ Department of Clinical Genetics and Genomics Region Västra Götaland Sahlgrenska University Hospital Gothenburg Sweden

**Keywords:** BAF, BRD4, EWSR1‐FLI1, FUS‐DDIT3, Sarcoma, SWI/SNF

## Abstract

FET fusion oncoproteins containing one of the FET (FUS, EWSR1, TAF15) family proteins juxtaposed to alternative transcription‐factor partners are characteristic of more than 20 types of sarcoma and leukaemia. FET oncoproteins bind to the SWI/SNF chromatin remodelling complex, which exists in three subtypes: cBAF, PBAF and GBAF/ncBAF. We used comprehensive biochemical analysis to characterize the interactions between FET oncoproteins, SWI/SNF complexes and the transcriptional coactivator BRD4. Here, we report that FET oncoproteins bind all three main SWI/SNF subtypes cBAF, PBAF and GBAF, and that FET oncoproteins interact indirectly with BRD4 via their shared interaction partner SWI/SNF. Furthermore, chromatin immunoprecipitation sequencing and proteomic analysis showed that FET oncoproteins, SWI/SNF components and BRD4 co‐localize on chromatin and interact with mediator and RNA Polymerase II. Our results provide a possible molecular mechanism for the FET‐fusion‐induced oncogenic transcriptional profiles and may lead to novel therapies targeting aberrant SWI/SNF complexes and/or BRD4 in FET‐fusion‐caused malignancies.

## Graphical abstract

FET oncoproteins are characteristic oncogenic drivers in different sarcomas. We show that the FET oncoproteins FUS‐DDIT3 and EWSR1‐FLI1 interact with the main SWI/SNF subtypes cBAF, PBAF and GBAF/ncBAF, as well as with the transcriptional coactivator BRD4 via the SWI/SNF complex. FET oncoproteins, SWI/SNF components and BRD4 co‐localize on chromatin, potentially together with mediator and RNA polymerase II. Our results provide a possible oncogenic mechanism in FET sarcoma.

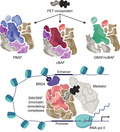

**Abbreviations**
BET: bromodomain and extra‐terminal motif
BWA: Burrows–Wheeler aligner
cBAF: canonical BAF complex
ChIP‐seq: chromatin immunoprecipitation sequencing
Co‐IP: co‐immunoprecipitation
EWS: Ewing sarcoma
EWSR1: Ewing sarcoma breakpoint region 1
FET: FUS; EWSR1 and TAF15
FET‐FOP: FET fusion oncoprotein
FUS: fused in sarcoma
GBAF: GLTSCR1/1L‐BAF complex
GEO: gene expression omnibus database
MLS: myxoid liposarcoma
MLSRC: myxoid liposarcoma high‐grade round‐cell type
MS: mass spectrometry
ncBAF: non‐canonical BAF complex
PBAF: polybromo BAF complex
PLAAC: prion‐like amino acid composition
PRC2: polycomb repressive complex 2
PRIDE: proteomics identifications database
PROTAC: proteolysis‐targeting chimera
PScore: phase separation propensity score
PTM: post‐translational modification
PVDF: polyvinylidene difluoride
QWB: quantitative western blot
SSE: sequential salt extraction
TAF15: TATA‐box binding protein associated factor 15
TSS: transcription start site

## 1 Introduction

**Fig. 1:**
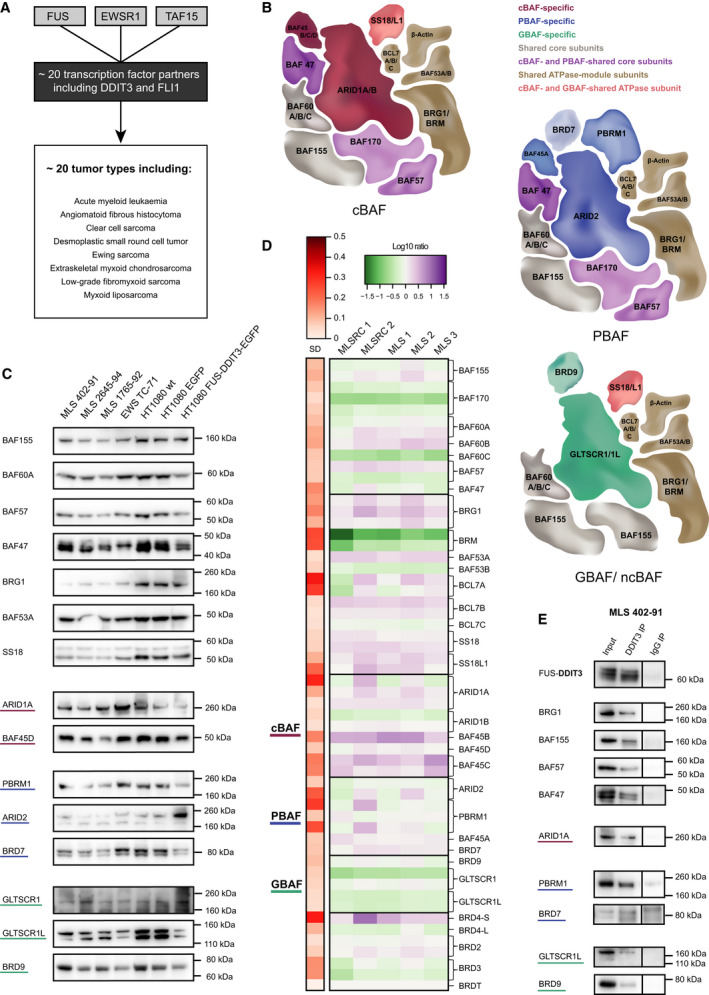
SWI/SNF subtypes and interactions with FET oncoproteins. (A) Schematic illustration of FET fusion oncoproteins that contain one of the FET proteins (FUS, EWSR1 or TAF15) together with a DNA‐binding transcription‐factor partner. FET oncoproteins are characteristic of many different subtypes of sarcoma and leukaemia, with a few examples listed. Additional FET‐FOPs are continuously being discovered. (B) Schematic illustration of the three SWI/SNF subtypes: cBAF (canonical BAF), PBAF (polybromo BAF) and GBAF/ncBAF (GLTSCR1/L BAF, non‐canonical BAF). Note that some SWI/SNF components are represented by several paralogs, e.g. BAF60A/B/C that are mutually exclusive, and some subunits are unique to one (or two) SWI/SNF subtypes. The reported compositions differ slightly between studies, possibly due to the cell types studied, extraction and purification protocols, and analytical approach. (C) Western blot of 10 µg nuclear extracts (extracted in 500 mm KCl) visualizing SWI/SNF components in myxoid liposarcoma (MLS 402‐91, 2645‐94 and 1765‐92), Ewing sarcoma (EWS TC‐71) and HT1080 fibrosarcoma (wt, EGFP or FUS‐DDIT3‐EGFP) cells using antibodies against core components (BAF155, BAF60A, BAF57 and BAF47), ATPase module components (BRG1, BAF53A and SS18), cBAF (ARID1A and BAF45D), PBAF (PBRM1, ARID2 and BRD7) and GBAF (GLTSCR1, GLTSCR1L and BRD9). Loading controls with all blots are shown in Fig. S1. (D) Heatmap visualization of gene expression levels for SWI/SNF components in five myxoid liposarcoma tumours (two high‐grade MLS round‐cell type, MLSRC, and three low‐grade MLS) compared to HT1080, from cDNA microarray analysis. In some cases, two or more probes against the same gene were used, shown as separate rows. Gene expression levels are visualized by the normalized log10 ratios in green (less than HT1080) and purple (more than HT1080), and variation in gene expression (standard deviation, SD) is visualized in red. (E) Western blot analysis of DDIT3‐biotin immunoprecipitated (IP) nuclear extracts of MLS 402‐91, visualizing successful co‐IP of the SWI/SNF complex (BRG1, BAF155, BAF57 and BAF47) and the three subtypes: cBAF (ARID1A), PBAF (PBRM1 and BRD7) and GBAF (GLTSCR1L and BRD9) using a direct IP approach. Maximum amount of eluate and around 5% of input was loaded on the gel. All samples were run on the same gel and exposed together. Separating black lines indicate that IgG samples were from a different part of the gel with adjoining lanes not shown. Source data for all WBs (full membranes) are available as supporting information.

Fusion oncogenes consisting of FET family genes *FUS*, *EWSR1*, *TAF15* (Fused in sarcoma, Ewing sarcoma breakpoint region 1, TATA‐box binding protein associated factor 15) as 5’‐partners and alternative transcription factor‐coding genes as 3’‐partners are characteristic of more than 20 types of sarcoma and leukaemia [1], including myxoid liposarcoma (MLS) and Ewing sarcoma (EWS) (hereby also called FET sarcoma; Fig 1A). FET fusion oncoproteins (FET‐FOPs) invariably contain the N‐terminal domains of the FET partners juxtaposed to DNA‐binding parts of the transcription‐factor partners. While the molecular function of the DNA‐binding domains is obvious, the role of the FET‐derived N‐terminal parts was for long enigmatic. However, we recently identified the SWI/SNF (also known as BAF) chromatin remodelling complex as the major binding partner to the FET‐FOPs and that the highly disordered prion‐like N‐terminal domains of FET proteins mediate this interaction [2]. FET oncoproteins affect the function of the SWI/SNF complex and its interaction with chromatin, potentially via liquid‐liquid phase separation mediated by the FET N‐terminal domain [2, 3], but exactly how this contributes to malignancy remains to be elucidated.

The SWI/SNF chromatin remodelling complexes consist of around 15 distinct core proteins, some of which exist in several alternative paralogs. The combined assembly of the different subunits leads to a vast variety of complexes with over 1000 theoretical combinations [4]. A main function of SWI/SNF chromatin remodelling complexes is ATP‐dependent reorganization of nucleosomes to modulate DNA accessibility [5]. Gene expression is also regulated via antagonistic interactions between the SWI/SNF complex and Polycomb repressive complex 2 (PRC2) [5, 6, 7]. Three main types of SWI/SNF complexes (Fig. 1B) have been described based on their distinct composition and biochemical properties; the more abundant canonical BAF complex (cBAF), the larger and less abundant polybromo BAF complex (PBAF) and the recently discovered smaller SWI/SNF subtype, called GLTSCR1/1L‐BAF (GBAF) [8] or non‐canonical BAF (ncBAF) [4]. All SWI/SNF complexes contain one of the ATPase subunits BRG1 or BRM, and the structural subunits BAF155 and BAF170. Other core components are BAF47, BAF57, BAF60A/B/C as well as the ATPase module; containing BAF53A/B, BCL7A/B/C, β‐actin, and sometimes SS18/L1. The cBAF complex uniquely contains ARID1A/B and BAF45B/C/D, while PBAF exclusively contains ARID2, PBRM1, BAF45A and BRD7 [9, 10, 11]. GBAF is defined by the two mutually exclusive paralogs GLTSCR1 and GLTSCR1L, contains BRD9 and the ATPase module but not the core components BAF47, BAF57, BAF170 nor any of the ARID or BAF45 paralogs [4, 8, 12]. The three main SWI/SNF subtypes have both shared and individual genomic binding patterns; cBAF primarily localizes to enhancers, PBAF is found at promoters and gene bodies, and GBAF mainly localizes to CTCF sites and promoters [12, 13, 14, 15]. The distinct functional modules of subtype‐specific SWI/SNF subunits, such as DNA‐ and chromatin‐binding motifs, contribute to the diverse genomic binding capacity and functions of the main SWI/SNF subtypes. The different SWI/SNF complexes are important in determining cell identity and cell‐type specific gene expression profiles [16, 17], but the exact roles of variant complexes as well as the functional contribution of each subunit has not been determined. Furthermore, it is not known which SWI/SNF subtypes are targeted by the FET‐FOPs and if these interactions affect the complex compositions and functions.

The bromodomain protein BRD4 has been reported to interact with FUS‐DDIT3 [18] and EWSR1‐FLI1/ERG [19], the specific FET‐FOPs in MLS and EWS, respectively, and plays a role in the aberrant gene expression induced by these oncoproteins. BRD4 belongs to the BET (bromodomain and extra‐terminal motif) family together with BRD2 and BRD3. They contain two bromodomains that recognize and bind acetylated histone tails, such as H3K27Ac and H4K16Ac. BRD4 regulates both enhancer activity and transcription [19], potentially via its interactions with transcription elongation factor complex P‐TEFb and RNA polymerase II [20]. Furthermore, BRD4 binding is considered as a mark for super‐enhancers and have demonstrated differential genomic binding patterns dependent on cell type and differentiation state [21]. BRD4 was recently reported to associate with the SWI/SNF complex [22, 23], especially to GLTSCR1/1L/BRD9 in the GBAF complex [8, 13, 24]. However, the interactions between BRD4 and SWI/SNF as well as FET‐FOPs are not clearly defined, and how BRD4 contributes to oncogenesis in FET‐FOP‐caused cancer is still unknown.

The aim of this study was to characterize the interactions between FET oncoproteins (FUS‐DDIT3 and EWSR1‐FLI1) and the different SWI/SNF complexes as well as the transcriptional coactivator BRD4, to investigate their roles in FET sarcoma. First, we used global gene expression analysis to evaluate the expression of SWI/SNF components in FET sarcoma cells and tumours. Then, we used comprehensive biochemical and proteomic analysis of nuclear proteins, including co‐immunoprecipitation (co‐IP), quantitative western blot (QWB) and sequential salt extraction (SSE), to further characterize the interactions between FET‐FOPs, SWI/SNF complexes and BRD4. We used chromatin immunoprecipitation sequencing (ChIP‐seq) to define their genome binding patterns, immunofluorescence analysis to analyze co‐localization and then evaluated the impact of BRD4 inhibition or degradation on FET sarcoma cells. Lastly, we evaluated the FET‐FOP interactomes, including their phase separation propensity.

## 2 Materials and methods

### 2.1 Cell culture

Myxoid liposarcoma cell lines (MLS 1765‐92, 402‐91 and 2645‐94) were previously established by us from MLS tumour tissues as described for MLS 402‐91 cells [25]. The fibrosarcoma cell line HT1080 [26] was obtained from ATCC (CCL‐121; Manassas, VA, USA) and the Ewing sarcoma cell line (EWS TC‐71) was a kind gift from Dr. Katia Scotlandi (University of Bologna, Italy). The MLS and HT1080 cell lines were cultured in RPMI 1640 GlutaMAX supplemented with 5% foetal bovine serum, and the EWS cells were cultured in IMDM GlutaMAX with 10% foetal bovine serum. Human F470 fibroblasts were cultured in RPMI with 10% foetal bovine serum. Culture media was supplemented with 100 U·mL^−1^ penicillin and 100 μg·mL^−1^ streptomycin. All media and supplements were obtained from Gibco (Thermo Fisher Scientific, Waltham, MA, USA). Cells were maintained at 37 °C in 5% CO_2_. The unique fusion oncogene content of the sarcoma cell lines used in the present study was previously confirmed by RT‐PCR analysis [2]. All cell lines were routinely screened for mycoplasma infections.

### 2.2 Transfection

Transient transfection of HT1080 cells with the pEGFP‐N1 expression vector (empty or containing the fusion oncogenes *FUS‐DDIT3* [27] or *EWSR1‐FLI1* [2]) was done using FuGENE® 6 Transfection Reagent (Promega, Madison, WI, USA) according to the manufacturer’s recommendations, scaled‐up to 75 cm^2^ culture flasks. Cells were transfected at ~ 60% confluency using a transfection reagent (µL) to DNA (µg) ratio of 3 : 1. Nuclear protein extraction was performed 24 h after transfection. Stable clones of HT1080 expressing FUS‐DDIT3‐EGFP, EWSR1‐FLI1‐EGFP and EGFP were established as described elsewhere [27] and maintained by the addition of 500 µg·mL^−1^ G418 (#11811‐064, Gibco, Thermo Fisher Scientific).

### 2.3 Microarray analysis of MLS tumours

Total RNA was extracted using TRIzol™ reagent (Thermo Fisher Scientific) from frozen tumour tissues of five myxoid liposarcoma (MLS) tumours, of which two were high‐grade round‐cell type (MLSRC). Tumour and reference (HT1080 wt) RNA were labelled with Cy5 and Cy3 nucleotides during cDNA synthesis followed by microarray analysis as previously described [28]. Equal quantities of labelled cDNA and reference cDNA were hybridized to Agilent Whole Human Genome Microarray 4x44K G4112F microarrays (Agilent Technologies, Santa Clara, CA, USA). The arrays were scanned using an Agilent G2565CA microarray scanner and analysis was performed with Agilent’s Feature Extraction 10.4 image analysis software. Normalized log10 ratios were used to compare the expression levels of SWI/SNF components in MLS tumour samples. Raw and normalized microarray data generated during the current study were deposited according to MIAME standards in NCBI's Gene Expression Omnibus (GEO) with accession number GSE167270 [https://www.ncbi.nlm.nih.gov/geo/query/acc.cgi?acc=GSE167270]. Frozen MLS tumours were treated in accordance with the Declaration of Helsinki, including written consent, as approved by the Regional ethical review board (133‐11; Gothenburg, Sweden).

### 2.4 RNA sequencing analysis

Expression levels of SWI/SNF components were analyzed using normalized RNA sequencing data for HT1080 wt, HT1080 EGFP and HT1080 cells with stable ectopic expression of FET‐FOPs FUS‐DDIT3‐EGFP or EWSR1‐FLI1‐EGFP from a previous study [2] (accession number GSE125941) and publicly available RNA sequencing data sets for the Ewing sarcoma cell lines TC‐71 and A673 transfected with siRNA targeting EWSR1‐FLI1 (accession number GSE132966) and human mesenchymal stem cells with EWSR1‐FLI1 expression (accession number GSE94277) retrieved from NCBI’s Gene Expression Omnibus (GEO) database [29] as counts matrices. Normalized counts and differential expression of SWI/SNF components were analyzed using the R package DESeq2 (version 1.29.1), based on shrink estimation for dispersion and fold‐change using a negative binomial distribution model [30].

### 2.5 Mass spectrometry analysis

To determine the composition of FET‐FOP‐bound SWI/SNF complexes such as SWI/SNF subtype‐specific components, and FET oncoprotein interactomes, mass spectrometry proteomics data identifying proteins in DDIT3 eluates (FUS‐DDIT3 in MLS 402‐91; DDIT3‐biotin antibody, NB600‐1335B, Novus Biologicals, Littleton, CO, USA) and FLI1 eluates (EWSR1‐FLI1 in EWS TC‐71; FLI1‐biotin, 246159‐biotin, US Biologicals, Salem, MA, USA) from a previous study [2] was used (PXD012680; deposited at the ProteomeXchange Consortium via the PRoteomics IDEntifications database (PRIDE) [https://www.ebi.ac.uk/pride/archive/]).

### 2.6 Cell viability assay and BRD4 inhibition

To determine the cytotoxic effect of BRD4 inhibition on MLS, EWS and HT1080 tumour cell lines as well as fibroblasts (F470) *in vitro*, cells were treated with 6.4 nm–10 µm of the dual‐bromodomain inhibitor AZD5153 (AstraZeneca, Cambridge, Great Britain; inhibits both bromodomains) followed by alamarBlue cell viability assay, according to the manufacturer’s protocol (Thermo Fisher Scientific) using two biological replicates. AZD5153 was dissolved in DMSO and aliquoted in stock solutions of 150 mm and stored at −80 °C. For cell viability assays, working solutions of 1 mm were prepared in PBS. Before treatment, cells were seeded in 100 µL RPMI GlutaMAX medium in Eppendorf 96‐well cell culture plates at cell densities of 1500‐3000 cells·well^−1^, depending on the proliferation rate. PBS was added in the outer wells and in the chambers between cells to diminish evaporation during incubation and reduce edge effects. After 24 h, cells were treated for 72 h by addition of 100 µL AZD5153 using a five‐fold dilution series (six replicates per dose; final concentrations 6.4 nm – 10 µm). The DMSO concentration was negligible (0.0078%), and therefore, no DMSO vehicle control was used. The cells were then incubated with 20 µL of alamarBlue for 4 h. The absorbance was measured with an excitation frequency of 544 nm and an emission frequency of 615 nm using a Wallac Victor3 1420 Multilabel Counter (PerkinElmer, Waltham, MA, USA). Dose‐response curves were generated (two biological replicates; six technical replicates each) using graphpad Prism by performing background subtraction for medium control and normalizing treated samples to untreated controls (version 9.0.0, Graphpad, San Diego, CA, USA). IC50 values were estimated using normalized data points fitted into sigmoidal non‐linear regression curves.

To determine the effect of BRD4 inhibition on FET oncoprotein and SWI/SNF interactions, MLS 402‐91 cells were expanded to six 15 cm petri dishes. Three of the petri dishes were treated with 500 nm AZD5153 for 24 h before nuclear protein extraction with the remaining three as control.

The BRD4 PROTAC degrader ARV‐825 (S8297, Selleckchem, Houston, TX, USA) was dissolved in DMSO, aliquoted in stock solutions of 100 mm and stored at −80 °C. A working solution of ARV‐825 was prepared in ultrapure water to reach a concentration of 100 µm. Cell viability assays were performed with 0.06‐1000 nm of ARV‐825 as described above. To validate the BRD4 degradation, MLS 402‐91 cells were seeded in Eppendorf 6‐well plates at a cell density of 250 000 cells·well^−1^ in 1.5 mL RPMI medium, with PBS between the wells. The next day, cells were treated with ARV‐825 at a final concentration of 200, 600, 1000 or 5000 nm for 3 h or 6 h. After incubation, cells were harvested by whole‐cell extraction. In brief, cells were washed in ice cold PBS (Thermo Fisher Scientific), followed by scraping in RIPA lysis buffer supplemented with 1x Halt Protease and Phosphatase Inhibitor Cocktail and 5 mm EDTA (all from Thermo Fisher Scientific). The cell lysate was resuspended and incubated for 5 min on ice twice, prior to 10 min of sonication at 30 s on/off intervals using Bioruptor® Pico sonication device (Diagenode, Liege, Belgium) to disrupt viscous nucleic acids. The cell lysate was then centrifuged at 14 000 rcf for 10 min at 4 °C. Protein concentration was determined using DC Protein Assay (Bio‐Rad Laboratories, Hercules, CA, USA), following the manufacturer’s instructions.

### 2.7 Nuclear protein extraction and sequential salt extraction

Cells were seeded on at least two T75 cell culture flasks or 15 cm petri dishes. Half the medium was replaced the day before nuclear protein extraction to ensure healthy cells. Collection of cells were done by scraping in PBS followed by centrifugation at 450 rcf for 10 min at 4 °C. The volume of the packed cell pellet was estimated and all volumes were adjusted to the amount of cells. First, the pellet was resuspended in five packed cell volumes of hypotonic lysis buffer (10 mm KCl, 10 mm Tris pH 7.5, 1.5 mm MgCl_2_; Ambion, Thermo Fisher Scientific) supplemented with 1 mm DTT (Sigma‐Aldrich, Merck, Darmstadt, Germany) and 1x Halt Protease Inhibitor Cocktail (Thermo Fisher Scientific). Cells were allowed to swell for 15 min on ice, and the supernatant was discarded after centrifugation at 400 rcf for 5 min at 4 °C. The cells were thereafter resuspended in two packed cell volumes hypotonic lysis buffer and treated with 5 U·mL^−1^ Benzonase (#71205, Merck Millipore, Merck) for 15 min on rotation at 4 °C before cell disruption by two (EWS cells) or five strokes of a syringe with a 27‐gauge needle. The cytoplasmic fraction was removed after centrifugation at 10 000 rcf for 20 min at 4 °C. The nuclear pellet was resuspended in two‐thirds packed cell volume high‐salt extraction buffer (0.42 m KCl, 10 mm Tris pH 7.5, 0.1 mm EDTA (Thermo Fisher Scientific), 10% glycerol (Merck Chemicals, Merck) supplemented with 1x halt protease inhibitor cocktail and gently agitated in an icebox for 30 min thereby equalizing the cell’s liquid interior with the added high‐salt extraction buffer resulting in an effective salt concentration around 250 mm KCl. The 250 mm KCl nuclear fraction was collected after centrifugation at 20 000 rcf for 5 min at 4 °C and diluted to 150 mm KCl salt concentration with dilution buffer (10 mm Tris pH 7.5, 0.1 mm EDTA, 10% glycerol, supplemented with 1x Halt Protease Inhibitor Cocktail), snap‐frozen on dry ice and stored at −80 °C. The protein concentration was measured using Bio‐rad DC protein assay.

For sequential salt extracts (SSE), the remaining nuclear pellet was resuspended and agitated for 30 min in an icebox using two‐thirds packed cell volumes of the same nuclear extraction buffer but with a higher salt concentration (0.6 m KCl, 10 mm Tris pH 7.5, 0.1 mm EDTA, 10% glycerol, supplemented with 1× Halt Protease Inhibitor Cocktail). After collection of the 500 mm KCl fraction, the nuclear pellet was resuspended a third time with 1.2 m KCl extraction buffer and the corresponding 1000 mm fraction was collected after 30 min incubation. Each fraction was collected after centrifugation at 20 000 rcf for 5 min at 4 °C and diluted to 150 mm salt concentration. We based our SSE experiments on the nuclear extraction protocol used for our immunoprecipitation experiments to compare the data with our current and previous biochemical investigations [2]. The caveat is that it includes Benzonase treatment during cell disruption to degrade nucleic acids and increases the recovery of nuclear proteins, which means that accessible DNA, for example in open chromatin regions, is digested and nuclear proteins including histones can be released. Amounts of nuclear fractions, corresponding to equal initial volume of each salt fraction, were evaluated with western blot to enable direct comparisons of the amounts of protein extracted in each fraction. Statistical analysis of SSE binding profiles were done using graphpad Prism software by 2‐way repeated measurement ANOVA, where the difference between proteins was evaluated based on the *P*‐value for the interaction term of proteins and concentrations, with the null hypothesis that any difference between proteins is identical at all concentrations. Bonferroni adjustments were applied for multiple comparisons.

To analyze SWI/SNF components and BRD4 isoforms, 500 mm KCl salt extracts were extracted from MLS, EWS and HT1080 cells using the nuclear extraction protocol, but nuclear lysates were extracted directly using the high‐salt extraction buffer with 870 mm KCl (instead of 420 mm).

### 2.8 Immunoprecipitation

An indirect immunoprecipitation (IP) protocol was used to pulldown the SWI/SNF complex as described below. Nuclear extracts (50 µg) were diluted to 250 µL with IP wash buffer (150 mm KCL, 10 mm Tris pH 7.5, 0.1 mm EDTA, 10% glycerol, supplemented with 1x protease inhibitor) and incubated overnight with gentle rotation at 4 °C with 5 µg antibody; either BAF155‐biotin (213471‐Biotin, US Biologicals) or BRG1‐biotin (ab200911, Abcam, Cambridge, UK), with normal mouse IgG‐biotin (sc‐2762, Santa Cruz Biotechnology, Dallas, TX, USA) or normal rabbit IgG‐biotin (sc‐2763, Santa Cruz Biotechnology) as negative control. The next day, 37.5 µL Streptavidin‐labelled magnetic beads (Dynabeads Myone Streptavidin T1, Thermo Fisher Scientific) per reaction were blocked for approximately 30 min in 1x Rotiblock (Carl Roth, Karsruhe, Germany) diluted in IP wash buffer, followed by three washes with IP wash buffer. The nuclear extract/antibody mix was thereafter added to the washed beads and incubated for 2 h at 4 °C with gentle rotation. The non‐bound fraction was collected, and the beads were washed 3 times for 5 min on gentle rotation with IP wash buffer. Captured protein complexes were eluted twice with 25 µL 2x NuPAGE LDS sample buffer with 10% NuPAGE sample reducing agent (Thermo Fisher Scientific) at 90 °C, 500 r.p.m. for 10 min, and pooled. Non‐bound samples were mixed with 4× NuPAGE LDS Sample buffer. All samples were stored at −20 °C.

For the DDIT3‐biotin IP, a direct immunoprecipitation approach using the same amounts, products and buffers as above was performed. However, streptavidin magnetic beads were incubated with DDIT3‐biotin antibody (NB600‐1335B, Novus Biologicals) for 30 min in block buffer on gentle rotation at 4 °C, and beads were washed three times so that the remaining unbound antibody was washed away, before overnight incubation with the nuclear extract. Captured protein complexes were moved to new 1.5 mL tubes before elution to reduce background.

In Quantitative Western blot (QWB) experiments, input and bound samples (eluates) were diluted relative to non‐bound (in IP wash buffer and ultrapure water respectively) considering dilutions during IP. This allows direct quantification of the fraction of bound and non‐bound protein (see also Fig. S3A). For regular WB experiments, the maximum amount of bound and non‐bound samples were loaded on the gel while diluting the input relative to non‐bound (around 5% of input).

### 2.9 Western blot

Proteins from sequential nuclear extracts, 500 mm nuclear extracts or IP‐samples were size‐separated with gel electrophoresis using the Novex NuPAGE system (Thermo Fisher Scientific). All samples were mixed to a final concentration of 1× NuPAGE LDS sample buffer and 10% NuPAGE sample reducing agent, denatured at 70 °C for 10 min (or 95 °C if histones were evaluated) and separated on NuPAGE 4‐12% Bis‐Tris gels. Proteins were transferred to PVDF membranes (polyvinylidene difluoride, 0.45 µm, Thermo Fisher Scientific) by wet blot and blocked with 5% skim milk (Merck Chemicals, Merck) or 5% bovine serum albumin (Sigma‐Aldrich, Merck) in TBS‐T buffer (50 mm Tris‐HCl pH 6.8, 50 mm NaCl, 0.1% Tween 20; Sigma‐Aldrich, Merck). Membranes were incubated overnight at 4 °C with primary antibodies (Table S1) in block buffer. After washes in TBS‐T, membranes were incubated at room temperature for 1 h with anti‐mouse or anti‐rabbit HRP‐conjugated secondary antibody (32430 and 32460; Thermo Scientific). Protein amounts were detected via chemiluminescent signals captured by ImageQuant LAS 4000 mini or ImageQuant Amersham 800 (GE Healthcare, Chicago, IL, USA) after incubation with SuperSignal West Dura Extended Duration Substrate for nuclear extracts or SuperSignal West Femto Max Sensitivity Substrate (Thermo Scientific) for IP samples. On rare occasions, membrane pieces were stripped with ReBlot Plus (2504, Merck Millipore) during 15 min incubation in room temperature, and after verifying successful stripping, relabelled with another primary antibody. Bands were quantified using ImageJ (1.52A, National Institutes of Health, USA).

### 2.10 ChIP sequencing and analysis

Chromatin immunoprecipitation (ChIP) was performed using the iDeal ChIP‐seq kit for Transcription Factors (Diagenode) according to the manufacturer’s instructions. To cross‐link proteins with DNA, formaldehyde was mixed with fixation buffer (all buffers from kit, Diagenode) to a final concentration of 11%, then added to the medium of cultured MLS 402‐91 cells in a proportion of 1 : 10, and incubated with gentle shaking at room temperature for 15 min. The reaction was stopped by addition of glycine (1 : 10) during gentle shaking for 5 min at room temperature. Cells were collected on ice by scraping and were then lysed with lysis buffer followed by shearing buffer. Chromatin was sheared by sonication using Bioruptor Pico sonication device (Diagenode; 7 cycles, 30 s ON, 30 s OFF). Optimal shearing (100‐600 bp) was assessed with a fragment analyzer using the DNF‐474 HS NGS Fragment Kit (Agilent Technologies). ChIP‐grade antibodies BAF155 (5 µL, #11956, Cell signaling technology, Danvers, MA, USA), BRG1 (10 µL, #49360, Cell signaling technology) and DDIT3 (2.5 µL, #2895, Cell Signaling Technology) were mixed with prewashed magnetic beads (Diagenode) for approximately 2 h at 4 °C, then mixed with 250 µL of sheared chromatin (~ 4 million cells) and incubated under constant rotation at 4 °C overnight. The beads were washed with wash buffers and then incubated for 30 min at room temperature in the provided elution buffer. An input sample of 2.5 µL sheared chromatin was de‐crosslinked and purified the same way as ChIP‐samples. All samples were incubated overnight at 65 °C to de‐crosslink proteins and DNA. For DNA purification, IPure beads v2 (Diagenode) were used; the beads were washed, then resuspended with Buffer C and the purified immunoprecipitated DNA was kept at −20 °C for further analysis. The concentration was measured using Qubit™ dsDNA HS Assay Kit (Thermo Fisher Scientific).

Library preparation and sequencing was performed by Diagenode. In short, immunoprecipitated chromatin and input DNA was quantified using Qubit™ dsDNA HS Assay Kit (Thermo Fisher Scientific). Libraries were prepared using IP‐Star® Compact Automated System (Diagenode) using MicroPlex Library Preparation Kit v2 (12 indices; Diagenode). Optimal library amplification was assessed by qPCR using KAPA SYBR® FAST (Sigma‐Aldrich) on LightCycler® 96 System (Roche, Basel, Switzerland) and by Fragment Analyzer™ (Agilent Technologies) using the DNF‐474 High Sensitivity NGS Fragment Analysis Kit. Libraries were then purified (double‐size selected) using Agencourt® AMPure® XP (Beckman Coulter, Brea, CA, USA) and quantified using the Qubit™ dsDNA HS Assay. Finally, the fragment size was analyzed again by Fragment Analyzer™ (Agilent Technologies). Libraries were pooled and sequenced with Illumina technology with paired‐end reads of 50bp length.

ChIP‐seq data analysis was performed as following: the quality of the fastq reads was assessed using fastqc (Babraham Bioinformatics). Fastq reads were mapped to the hg38 reference genome using BWA (Burrows‐Wheeler Aligner) [31]. Peak calling was performed using MACS2 v.2.2.7.1 [32] for BAF155 (*n* = 1), BRG1 (*n* = 1) and DDIT3 (*n* = 2) samples with matching input control as background and using minimum FDR (q‐value) cutoff for peak detection set to 0.01. Peak annotation and analysis was performed in R v.3.6.1 using the ChIPseeker R‐package v1.20.0 [33]. ChIP‐seq data from Chen et al. [18] was downloaded from NCBI Sequence Read Archive using accession numbers SRR6792600 for DDIT3, SRR8207029 for BRD4 and SRR6792595 for input control, and data analysis was performed following the same steps as described above.

Overlapping binding sites (with at least one base overlap) were identified and Venn diagrams were plotted using the DiffBind R‐package, using the dba.plotVenn function with the bed‐files for DDIT3, BRG1 and BAF155 from our ChIP‐experiment, and BRG4 from the Chen et al. dataset. Peaks were annotated to nearby genes with the function annotatePeak, setting the parameter tssRegion to 3000 base pairs. *De novo* motif discovery analysis was performed with the programme findMotifsGenome.pl from HOMER v4.11 (Hypergeometric Optimization of Motif EnRichment) using a region size of 200bp [34]. Enrichment analysis of unique overlapping annotated genes was performed in R v4.0.2 using gene set collections (GO Biological processes, Hallmarks, Reactome, Genetic and Chemical perturbations) from molecular signature database (MSigDB) v7.0 by applying the enricher function from the clusterProfiler package [35]. The function was slightly modified to better mimic the settings used by the MSigDB enrichment web tool [36, 37] by defining the background as the total amount of human genes curated by the HUGO Gene Nomenclature Committee. The probability of overlap of the gene list of unique annotated peaks that bind FUS‐DDIT3 and at least one of the SWI/SNF components BRG1 and BAF155 in MLS 402‐91 with the list of genes significantly regulated by FUS‐DDIT3 in HT1080 cells (adjusted *P*‐value ≤ 0.05 and log2 fold change ≥ 1) was statistically evaluated using Fisher’s exact test, where all genes curated by the HUGO Gene Nomenclature Committee were used as the total number of genes. The ChIP‐seq data (raw fastq‐files) were deposited to the NCBI Sequence Read Archive with accession number PRJNA721591 [https://www.ncbi.nlm.nih.gov/sra].

### 2.11 Phase separation prediction (PScore)

We utilized a phase separation predictor developed by Vernon et al., to evaluate the phase separation propensity of FET‐FOPs and their interaction partners [38]. The algorithm is based on pi interaction frequency predicted from amino acid sequence. A PScore (phase separation propensity score) over 4 was used as a cutoff to classify proteins with ability to phase separate. It should be noted that the predictor is based on the probability of proteins to from non‐local planar pi‐pi contacts and other potential contributors to phase separation such as hydrophobic effect and electrostatics are not included. The python script for the final phase separation predictor (elife_phase_separation_predictor.py) was downloaded from DOI: https://doi.org/10.7554/eLife.31486.022. Protein sequences for MS‐identified interacting proteins were downloaded in FASTA format from UniProt (ftp.uniprot.org) for the human reference proteome (UP000005640) and used as input in the python script together with protein sequences of the fusion proteins FUS‐DDIT3 (type I: exons 7‐2, type II: 5‐2 and type 13‐2) and EWSR1‐FLI1 (type 1).

### 2.12 Immunofluorescence analysis

Immunofluorescence analysis was performed on cultured HT1080 cells transiently transfected with FUS‐DDIT3‐EGFP. Prior to transfection, the cells were seeded at 10 000 cells·well^−1^ into 4 well chamber slides (Millicell EZ‐slide, Merck Millipore) and incubated for 8 h to allow attachment of cells. Transfection was performed as previously described using the FUGENE 6 transfection reagent. The transfected cells were fixated with 4% formalin (Sigma‐Aldrich) 18 h after transfection, washed three times in PBS before incubation in methanol for 5 min at −20 °C. After being washed in PBS, the cells were blocked in PBS with 2% bovine serum albumin and 0.5% Tween 20 for 20 min before incubation with antibodies specific for BRG1 (Santa Cruz Biotechnology, mouse, sc‐17796) diluted 1 : 100, MED1 (Atlas antibodies, Stockholm, Sweden; rabbit, HPA0552818) diluted 1 : 50, and BRD4 (Abcam, rabbit, ab128874) diluted 1 : 100. The binding of primary antibodies was visualized with ALEXA555‐ and ALEXA594‐tagged goat anti‐mouse or goat anti‐rabbit antibodies diluted at 1 : 1000 (Invitrogen, Thermo Fisher scientific). The specificity of immunofluorescence signals was verified by controls including cells stained with only secondary antibody and with unmatched primary and secondary antibodies.

Z‐stack images of stained cell nuclei were captured for EGFP, ALEXA 555 and ALEXA 594 using a Zeiss LSM800 microscope and the Zeiss zen black software (Zeiss, Oberkochen, Germany). The fiji/imagej software package was used for image analysis [39] using the plugin JACoP [40] for co‐localization analysis.

## 3 Results

### 3.1 Components of cBAF, PBAF and GBAF complexes are expressed in FET sarcoma cells

Expression of SWI/SNF genes, especially subunit paralogs, varies between cell types. We therefore evaluated the protein expression of SWI/SNF components (and FET‐FOPs) in nuclear extracts from different sarcoma cell lines: myxoid liposarcoma (MLS 402‐91, 2645‐94 and 1765‐92), Ewing sarcoma (EWS TC‐71) and fibrosarcoma (HT1080 wildtype and HT1080 with stable ectopic expression of EGFP or FUS‐DDIT3‐EGFP) using WB analysis (Fig. 1C and Fig. S1A,B). SWI/SNF subunits specific for cBAF, PBAF and GBAF were expressed in sarcoma cells together with core components. Most SWI/SNF components showed lower expression in FET sarcomas compared to HT1080 cells, but no complete loss of any subunit was observed. Moreover, the WB analysis indicated that ectopic expression of FUS‐DDIT3 in HT1080 altered the expression levels to levels in FET sarcoma cells for several SWI/SNF components (both core and PBAF‐, GBAF‐specific components).

We then analyzed mRNA expression of SWI/SNF components in five MLS tumours using global mRNA expression analysis; most SWI/SNF components were expressed at similar levels compared to the HT1080 cell line (Fig. 1D). However, in MLS tumour tissue, there was slightly higher expression of cBAF‐specific components and lower expression of GBAF‐specific components compared to HT1080. The two high‐grade MLS tumours (round‐cell‐type) did not separate from the low‐grade MLS tumours based on expression of SWI/SNF components. Furthermore, stable ectopic expression of FET‐FOPs FUS‐DDIT3 and EWSR1‐FLI1 in HT1080 cells did not substantially change the mRNA expression of any SWI/SNF component (Fig. S1C). To corroborate this, we utilized publicly available mRNA expression data for Ewing cell lines A763 and TC‐71 with siRNA‐induced EWSR1‐FLI1 knockdown and mesenchymal stem cells with ectopic EWSR1‐FLI1 expression, and observed no clear differences in expression levels for SWI/SNF components (Fig. S1D).

### 3.2 FET oncoproteins interact with all three main SWI/SNF subtypes cBAF, PBAF and GBAF

FET oncoproteins interact with the SWI/SNF complex [2, 3]. To determine which of the main SWI/SNF subtypes cBAF, PBAF and GBAF that interact with FET‐FOPs, we utilized the dataset of mass spectrometry (MS)‐identified proteins that co‐immunoprecipitated with FET‐FOPs in MLS 402‐91 and EWS TC‐71 [2]. The MS data revealed that FET‐FOP‐bound complexes contain cBAF‐specific components such as ARID1A/1B, PBAF‐specific components such as ARID2 and PBRM1, and GBAF‐specific components BRD9 and GLTSCR1L (Table 1). However, the MS analysis did not identify BRD7 (PBAF‐specific) nor GLTSCR1 (GBAF‐specific) in the FET‐FOP‐bound SWI/SNF complexes. To verify whether FET‐FOPs bind to all main SWI/SNF subtypes, we performed a DDIT3 IP and immunoprecipitated FUS‐DDIT3 in MLS 402‐91 nuclear extracts. Since the stress‐induced endogenous DDIT3 protein is not expressed in normal growth conditions, all analysis with a DDIT3 antibody only targets the oncoprotein. Western blot analysis verified that SWI/SNF complexes co‐immunoprecipitated with FUS‐DDIT3, as indicated by the presence of BRG1, BAF155, BAF57 and BAF47 in the DDIT3 bound fraction (Fig. 1E). Further investigation revealed the presence of ARID1A (cBAF), PBRM1 and, although weaker, BRD7 (PBAF), GLTSCR1L and BRD9 (GBAF) in the co‐IP, demonstrating that FET oncoproteins FUS‐DDIT3 and EWSR1‐FLI1 interact with all three SWI/SNF subtypes (Fig. 1E).

**Table 1:** Composition of FET‐FOP‐bound SWI/SNF complexes.

Protein	Alternative name	SWI/SNF subtype	DDIT3 Co‐IP	FLI1 Co‐IP
BAF155	**SMARCC1**	All, core module	MS	MS
BAF60A	**SMARCD1**		MS	MS
BAF60B	**SMARCD2**		MS	MS
BAF60C	**SMARCD3**			MS
BAF170	**SMARCC2**	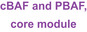	MS	MS
BAF57	**SMARCE1**		MS	MS
BAF47	**SMARCB1**		MS	MS
BRG1	**SMARCA4**	All, ATPase	MS	MS
BRM	**SMARCA2**			MS
BAF53A	**ACTL6A**	All, ATPase module	MS	MS
BAF53B	**ACTL6B**			
β‐ACTIN	**ACTB**		MS	MS
**BCL7A**				MS
**BCL7B**				MS
**BCL7C**				
**SS18**	SSXT	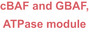	MS	
**SS18L1**	CREST		MS	
**ARID1A**	BAF250A	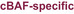	MS	MS
**ARID1B**	BAF250B		MS	MS
BAF45B	**DPF1**			
BAF45C	**DPF3**		MS	
BAF45D	**DPF2**		MS	MS
**ARID2**	BAF200	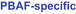	MS	MS
**PBRM1**	BAF180		MS	MS
BAF45A	**PHF10**			MS
**BRD7**				
**BRD9**		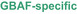	MS	
GLTSCR1	**BICRA**			
GLTSCR1L	**BICRAL**			MS
**BRD4**		SWI/SNF‐associated	MS	MS

Summary of SWI/SNF components in the bound fraction after DDIT3 Co‐IP of FUS‐DDIT3 in MLS 402‐91 and after FLI1 Co‐IP of EWSR1‐FLI1 in EWS TC‐71 as verified by mass spectrometry (MS). Note that some SWI/SNF components are represented by several paralogs, e.g. BAF60A‐C and some subunits are unique to one (or two) SWI/SNF subtypes such as **cBAF** , **PBAF** , or **GBAF/ncBAF** . Protein name in bold indicates the standardized nomenclature. Parts of this data were previously reported [2], the whole dataset is found in the PRIDE database with identifier PXD012680.

### 3.3 Diverse chromatin binding properties for FET oncoproteins and SWI/SNF subtypes

**Fig. 2:**
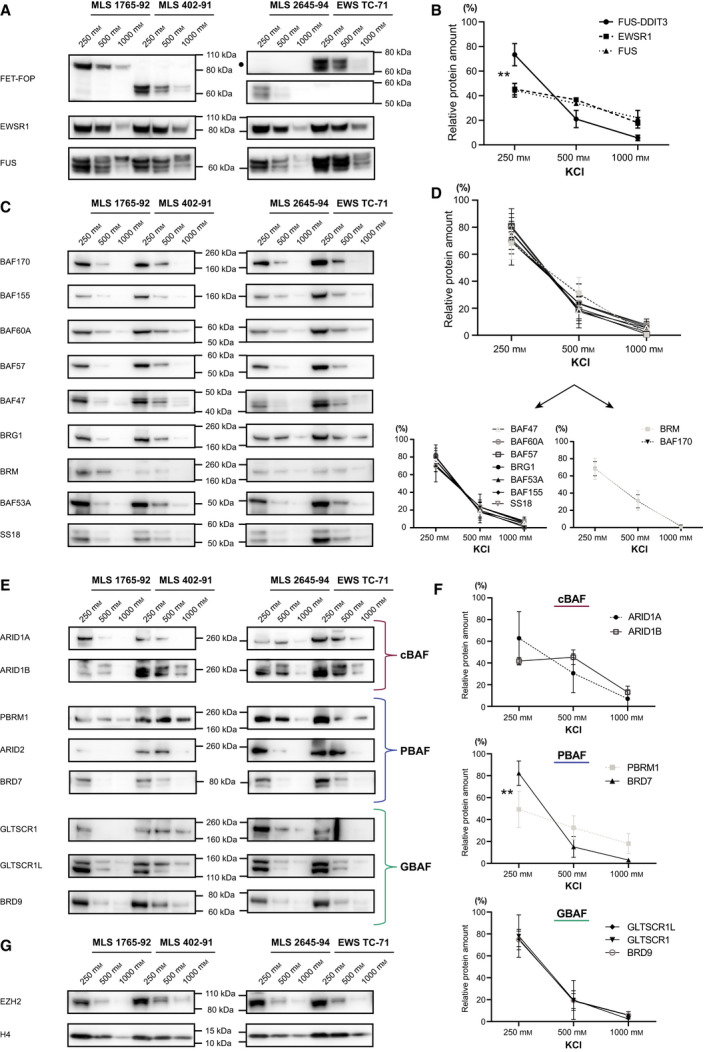
Sequential salt extraction analysis highlights diverse chromatin binding profiles. (A) Western blot (WB) analysis of MLS 1765‐92, 402‐91 and 2645‐94, and EWS TC‐71 sequential salt extraction (SSE) extracts visualizing FET‐FOPs FUS‐DDIT3 (DDIT3 antibody) or EWSR1‐FLI1 (● FLI1 antibody), as well as EWSR1 and FUS. (B) SSE binding profiles, visualizing the average amount of protein (*n* = 3) extracted in the different salt fractions in three MLS cell lines quantified from WB signals in A. FUS‐DDIT3 displayed a distinct binding profile compared to EWSR1 and FUS (adjusted *P*‐value 0.0016 and 0.0018, respectively). (C) Western blot analysis of MLS 1765‐92, 402‐91 and 2645‐94, and EWS TC‐71 SSE extracts visualizing SWI/SNF core subunits: BAF170, BAF155, BAF60A, BAF57, BAF47, BRG1, BRM, BAF53A and SS18. (D) SSE binding profiles for SWI/SNF core components (*n* = 4), quantified from WB signals in C. Two SWI/SNF core proteins had a slightly different binding profile as illustrated in the two smaller graphs. (E) Western blot analysis of MLS 1765‐92, 402‐91 and 2645‐94, and EWS TC‐71 SSE extracts visualizing SWI/SNF subtype‐specific subunits: ARID1A and ARID1B (cBAF), PBRM1, ARID2 and BRD7 (PBAF), and GLTSCR1, GLTSCR1L, and BRD9 (GBAF). (F) SSE binding profiles for cBAF‐, PBAF‐ and GBAF‐components (*n* = 4), quantified from WB signals in E. PBRM1 displayed a distinct binding profile compared to BRD7 (*P* = 0.0036). (G) Western blot analysis of MLS 1765‐92, 402‐91 and 2645‐94, and EWS TC‐71 SSE extracts visualizing PRC2‐component EZH2 and control Histone H4. (A, C, E, G) Loading WB: Nuclear SSE extracts (250 mm, 500 mm and 1000 mm), corresponding to equal initial volume of each salt fraction, were loaded on the gel to directly compare the amount of protein extracted in each fraction. (B, D, F) Binding profiles: Mean +/‐ SD (standard deviation) is shown, *n* = 3‐4. Statistical significance was determined by 2‐way ANOVA, ** *P* < 0.01. Detailed plots for each cell line is available in Fig. S2.

Next, we aimed to elucidate how SWI/SNF complexes and associated FET‐FOPs interact with chromatin in FET sarcoma cells. The properties of FET proteins and SWI/SNF subtypes, more specifically their binding strength to chromatin, was evaluated using a sequential salt extraction procedure, where nuclear proteins were extracted at increasing salt concentrations of 250 mm, 500 mm and 1000 mm KCl. Around 65% of total protein was extracted in the first 250 mm fraction followed by 25% and 10% in 500 mm and 1000 mm, respectively (Table S2). Western blot analysis of equal volumes of SSE extracts from three MLS cell lines (1765‐92, 402‐91 and 2645‐94) and one EWS cell line (TC‐71) revealed that normal FET proteins were released in higher salt concentrations and therefore bind stronger to chromatin than the FET oncoproteins (Fig. 2A and Fig. S2A). Combined SSE binding profiles from the three MLS cell lines, visualizing the amount of protein extracted in the different fractions, showed a significantly distinct binding pattern for FUS‐DDIT3 compared to the normal EWSR1 and FUS proteins (Fig. 2B). Some reports have indicated that FET‐FOPs bind to super‐enhancers, that is more accessible regions of chromatin, alone or together with SWI/SNF [3, 18, 19, 41]. This may explain why FET‐FOPs FUS‐DDIT3 and EWSR1‐FLI1 are more easily extracted from chromatin compared to the normal FET proteins.

Western blot analysis of SSE extracts revealed that a majority of the probed SWI/SNF proteins were extracted in the 250 mm KCl fraction, while very small amounts remained and were extracted in the 1000 mm KCl fraction (Fig. 2C‐D and Fig. S2B). Binding profiles showed similar binding patterns for most SWI/SNF core components, with minor differences for BRM and BAF170 (Fig. 2D). Analysis of SWI/SNF subtype‐specific components (Fig. 2E‐F and Fig. S2C) revealed that most of them shared a similar binding pattern with SWI/SNF core proteins (Fig. 2C‐D). Interestingly, PBAF‐specific components PBRM1 and BRD7 displayed significant differential binding strength; PBRM1 bound stronger to chromatin in MLS and EWS cells than BRD7 (Fig. 2F). Alternative interpretations of these data are that tightly chromatin‐bound PBAF complexes do not contain BRD7, that PBRM1 also binds chromatin with high binding strength as a free subunit [4] or that BRD7 binds as a monomer with lower binding strength than PBAF. The binding strength of PRC2 component EZH2 and histone H4 were evaluated as controls (Fig. 2G and Fig. S2D); EZH2 had a similar binding pattern as SWI/SNF components, while histone H4, as expected, required higher salt concentrations to be released from chromatin. In conclusion, PBAF complexes (potentially without BRD7) bind stronger to chromatin than the other two subtypes in FET sarcoma cells and FET oncoproteins have similar binding profiles as SWI/SNF components that are distinct from normal FET proteins. These results are supported by previous studies in other cell types showing that PBAF binds more strongly to chromatin than cBAF and GBAF [8, 42].

### 3.4 FET oncoproteins interact robustly with intact SWI/SNF complexes

**Fig. 3:**
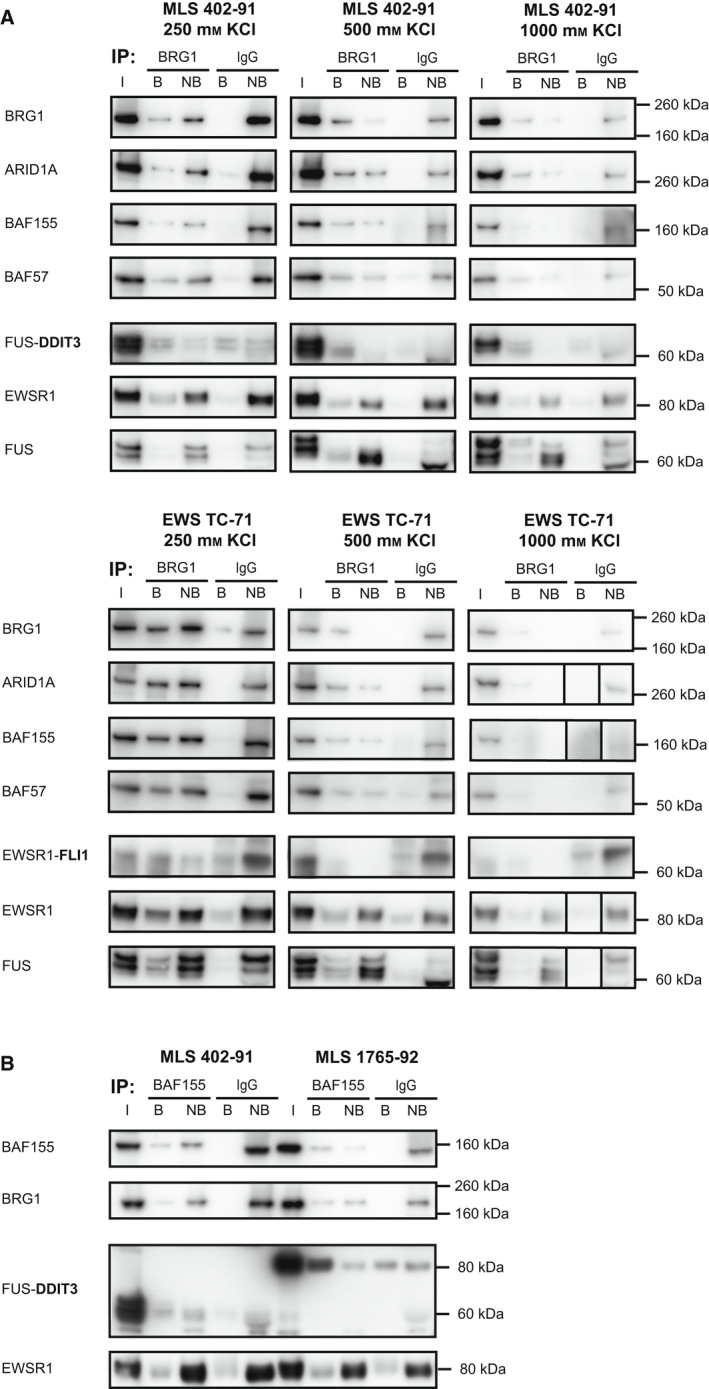
Robust interactions between the SWI/SNF complex and FET oncoproteins. (A) Quantitative western blot (QWB) analysis of BRG1‐biotin immunoprecipitated (IP) sequential salt extracts (250, 500 and 1000 mm KCl) in MLS 402‐91 and EWS TC‐71 sarcoma cell lines, visualizing SWI/SNF components (BRG1, ARID1A, BAF155 and BAF57), FET‐FOPs FUS‐DDIT3 and EWSR1‐FLI1 (antibody against C‐terminal partner) and normal FET proteins (EWSR1 and FUS). All IP samples for each cell line were evaluated on the same gel and bands for each antibody were treated equally although separate rectangles are shown for better visualization. Smaller rectangles indicate that the order of the samples were different and cut for visualization purposes. (B) Quantitative western blot analysis of BAF155‐biotin IP nuclear extracts of MLS 402‐91 and 1765‐92 sarcoma cell lines, visualizing SWI/SNF components (BAF155 and BRG1), FET‐FOP FUS‐DDIT3 and normal FET protein EWSR1. Another replicate for MLS 1765‐92 is shown in Fig. S3B. (A‐B) Loading IP‐QWB: Input (I) and eluate (bound, B) samples were diluted relative non‐bound (NB), so that B+NB=100%, as explained in Fig. S3A. Around 5% of input was loaded on the gel.

In the context of FET sarcoma, it is of interest to determine whether the SWI/SNF complex is intact in the presence of FET oncoproteins. To evaluate this, we performed IP with an antibody against the catalytic SWI/SNF subunit BRG1 using sequential salt nuclear extracts from MLS 402‐91 and EWS TC‐71. The SWI/SNF complex remained intact at 1000 mm salt as shown by co‐IP and quantitative western blot analysis (Fig. S3A) of ARID1A, BAF155 and BAF57 (Fig. 3A). Further analysis showed that FUS‐DDIT3 and EWSR1‐FLI1 co‐immunoprecipitated with SWI/SNF complexes, also in the 1000 mm KCl fraction i.e. with the fraction of complexes binding stronger to chromatin. Importantly, in this fraction the vast majority of FET‐FOPs were bound to SWI/SNF whereas only a small amount of the normal FET proteins (EWSR1 and FUS) co‐immunoprecipitated with the SWI/SNF complex (Fig. 3A).

A closer analysis of our BRG1 IP‐QWB experiments in the 250 mm salt fraction (Fig. 3A), revealed a recurrent trend that was even more apparent in our previous experiments [2] due to lower unspecific signals; the BRG1 IP pulled down only about half of the total amount of SWI/SNF complexes available in the nuclear extracts, whereas a majority of the FET‐FOPs were immunoprecipitated. These results suggest that the BRG1 antibody (ab200911) preferentially precipitates FET‐FOP‐bound SWI/SNF complexes. To evaluate this further, we immunoprecipitated SWI/SNF complexes in MLS cell lines (402‐91 and 1765‐92) targeting the core subunit BAF155 instead of BRG1, and a similar pattern as the BRG1 IP was observed (Fig. 3B and Fig. S3B). Taken together, these results suggest that FET oncoproteins bound to, or induced, SWI/SNF complex conformations that were preferentially accessible for immunoprecipitation.

### 3.5 Several BRD4 isoforms including post‐translational‐modified versions are expressed in FET sarcoma cells

**Fig. 4:**
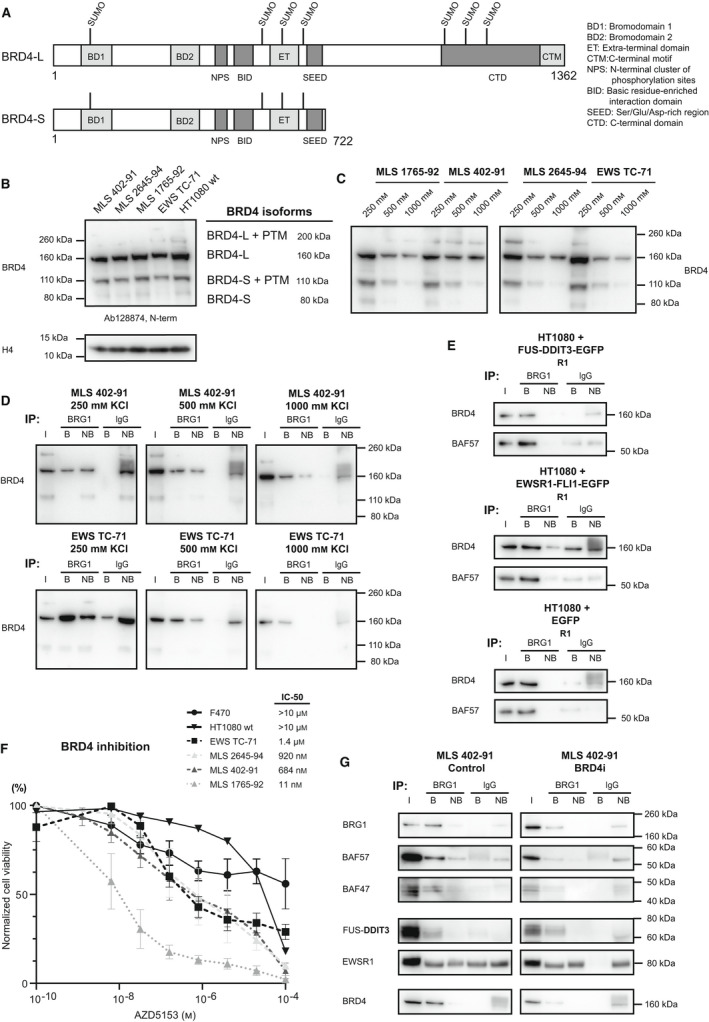
BRD4 expression, interactions and inhibition. (A) Schematic visualization of BRD4 long isoform (BRD4‐L) and short (BRD4‐S). Amino acid numbers and potential SUMO post‐translational modification sites (PTM) are indicated. (B) Western blot analysis (WB) of BRD4 isoforms in 10 µg nuclear extracts (extracted in 500 mm KCl) of MLS 402‐91, 2645‐94 and 1765‐92, EWS TC‐71 and HT1080 fibrosarcoma, using a BRD4 antibody (ab128874, N‐term, both isoforms). Analysis with two more BRD4 antibodies (C‐term) are shown in Fig. S4A. (C) Western blot analysis of MLS 1765‐92, 402‐91 and 2645‐94, and EWS TC‐71 SSE extracts visualizing short and long BRD4 isoforms (BRD4 antibody ab128874). Same samples and loading as in Fig. 2. (D) Quantitative western blot analysis of BRG1‐biotin immunoprecipitated (IP) sequential salt extracts (250, 500 and 1000 mm KCl) in MLS 402‐91 and EWS TC‐71, visualizing BRD4 isoform co‐IP (BRD4 antibody ab128874). Same samples and loading as in Fig. 3A. (E) Western blot analysis of BRG1‐biotin immunoprecipitated nuclear extracts of HT1080 fibrosarcoma cells transiently transfected (24h) with FUS‐DDIT3‐EGFP, EWSR1‐FLI1‐EGFP or EGFP control (R1) visualizing successful co‐IP of the SWI/SNF complex (BAF57) and BRD4. R2‐R3 are displayed in Fig. S4E. (F) Cell viability dose response curves of MLS cell lines 2645‐94, 402‐91 and 1765‐92, EWS TC‐71, HT1080 fibrosarcoma and fibroblasts (F470) after BRD4 inhibition (AZD5153, 72h). Mean +/‐ SD (standard deviation) is shown, *n* = 12 (2 biological, 6 technical replicates each). (G) Western blot analysis of BRG1‐biotin immunoprecipitated nuclear extracts of MLS 402‐91 control and BRD4‐inhibited cells (BRD4i, AZD5153, 24 h 500 nm) visualizing SWI/SNF components (BRG1, BAF57 and BAF47), FUS‐DDIT3, normal FET protein EWSR1 and BRD4. (E, G) Loading IP‐WB: Maximum amount of eluate (B) and non‐bound (NB) were loaded on the gel. Input (I) samples were diluted relative NB and around 5% of input was loaded.

The transcriptional coactivator BRD4 has been implicated in oncogenic transcription. Furthermore, recent studies in breast cancer and acute myeloid leukaemia reported opposite oncogenic functions of the short and long BRD4 isoform underlining the importance to distinguish between these isoforms [43, 44]. Therefore, we first evaluated the expression of BRD4 and its two main isoforms (Fig. 4A); the short (80 kDa) and the long isoform (150 kDa) in FET sarcoma tissues and cells. Our mRNA analysis showed that both the 80 and 150 kDa BRD4 isoforms were expressed in MLS tumour tissue (Fig. 1D). When evaluated on protein level in sarcoma cell lines, the three tested antibodies demonstrated substantial variations in the detection of BRD4 isoforms while there was no apparent difference between cell lines (Fig. 4B and Fig. S4A). In addition, the antibody targeting the N‐terminus of BRD4 that can detect both the short and long isoform (at 80 kDa and 160 kDa, respectively), also detected bands around 30 kDa larger than each isoform. These 110 kDa and 200 kDa bands are most likely the result of post‐translational modifications, such as SUMOylation [45]. Treatment with a BRD4 degrader (ARV‐825) that recruits BRD4 to the E3 ligase cereblon [46] specifically degraded the post‐translational‐modified BRD4 versions (Fig. S4B). This suggests a preferential degradation of isoforms marked by post‐translational modifications, potentially SUMOylation, by a proteolysis‐targeting chimera (PROTAC) degrader. Evaluation of sequential salt extracts revealed clear differences in chromatin binding strength for the different BRD4 versions; the unmodified 160 kDa isoform bound stronger than the other variants (Fig. 4C), indicating different functional roles and nuclear binding partners.

### 3.6 FET oncoproteins interact with BRD4 via the SWI/SNF complex

BRD4 has been reported to bind FET oncoproteins [18, 19] and the SWI/SNF complex [8, 24]. However, some proteomic studies have failed to identify BRD4 as a SWI/SNF interaction partner [13, 47], possibly indicating that this interaction is transient and weaker than those between SWI/SNF subunits and other binding partners. Our BRG1 IP of SSE nuclear extracts showed that BRD4 co‐immunoprecipitated with the SWI/SNF complex in MLS 402‐91 and EWS TC‐71 (Fig. 4D). BRD4 remained bound up to 1000 mm KCl, indicating a strong interaction in FET sarcoma cells. Interestingly, it was primarily the long BRD4 isoform at 160 kDa (Fig. 4D) and its post‐translational modified version at 200 kDa (Fig. S4C) that co‐immunoprecipitated with the SWI/SNF complex. The short modified BRD4 isoform at 110 kDa also precipitated with the SWI/SNF complex in EWS cells (Fig. 4D).

We verified BRD4 as a possible binding partner to FUS‐DDIT3 and EWSR1‐FLI1 using a direct DDIT3 IP and by further analyzing the FET‐FOP IP‐MS data (Fig. S4D and Table 1). We speculated that at least some of the interactions between BRD4 and FET‐FOPs are indirect and mediated by the SWI/SNF complex. Therefore, we evaluated the effect of FET oncoproteins on the BRD4–SWI/SNF interaction in HT1080 cells after transient expression of EGFP‐tagged FET‐FOPs or EGFP control followed by BRG1 IP. BRD4 co‐immunoprecipitated with BRG1 in HT1080 cells lacking FET‐FOPs, and the amount of precipitated BRD4 was not visibly affected by the presence of FUS‐DDIT3‐EGFP or EWSR1‐FLI1‐EGFP indicating that FET‐FOPs do not contribute to this interaction (Fig. 4E and Fig. S4E). Based on these results, we conclude that the co‐immunoprecipitation of BRD4 with the FET oncoproteins is mediated by the shared interaction with the SWI/SNF complex or, less likely, by a hitherto unknown shared interaction partner.

### 3.7 BRD4 inhibition reduces viability of FET sarcoma cells but does not disrupt the FUS‐DDIT3–SWI/SNF interaction

Targeting BRD4 in cancer has gained increased interest due to its multiple roles in transcriptional regulation. *In vitro* cell viability studies showed that cell lines from FET sarcomas (MLS 2645‐94, 402‐91 and 1765‐92, and EWS TC‐71) were more sensitive to BRD4 inhibition with the dual‐bromodomain inhibitor AZD5153 compared to HT1080 fibrosarcoma cells or normal fibroblasts (Fig. 4F). In addition, MLS cells (402‐91) were equally sensitive to BRD4 degradation by ARV‐825, with IC‐50 values in the nm ‐range (Fig. S4F). Based on these data, we tested if BRD4 inhibition affected the binding of FET‐FOPs to the SWI/SNF complex. Immunoprecipitation of BRG1 in BRD4‐inhibited cells showed no reduction of FUS‐DDIT3 Co‐IP compared to control cells, while a minor reduction of BRD4 bound to SWI/SNF was observed (Fig. 4G). Thus, we conclude that BRD4 bromodomain inhibition did not disrupt the FUS‐DDIT3 – SWI/SNF interaction.

### 3.8 FUS‐DDIT3, SWI/SNF components and BRD4 co‐localize on chromatin

**Fig. 5:**
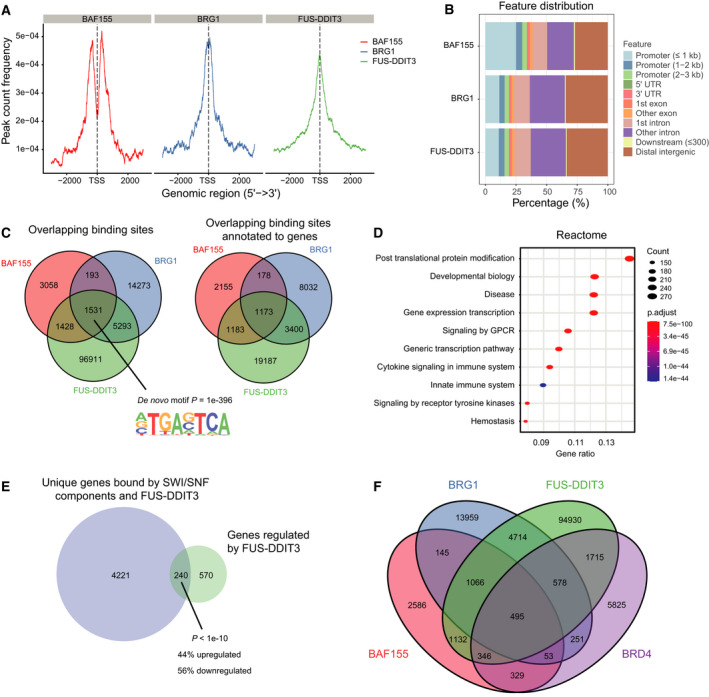
ChIP sequencing reveals co‐localization of FUS‐DDIT3, SWI/SNF components and BRD4. (A) ChIP‐seq peak profiles of BAF155, BRG1 and FUS‐DDIT3 in MLS 402‐91 ± 3 kb surrounding TSS (transcription start site). (B) Bar chart showing the genomic distribution of BAF155, BRG1 and FUS‐DDIT3 ChIP‐seq peaks in MLS 402‐91. The majority of peaks are located in promotors close to the TSS, in introns or distal intergenic sites. UTR: mRNA untranslated region. (C) Venn diagram depicting overlap of BAF155, BRG1 and FUS‐DDIT3 ChIP binding sites and annotated genes in MLS 402‐91. The same gene may appear multiple times. The top *de novo* motif identified with Homer motif discovery for the combined binding sites is shown. (D) Significantly enriched gene sets from the “Reactome” gene set collection using the 4461 unique genes bound by FUS‐DDIT3 and at least one SWI/SNF component. Top 10 based on gene ratio is shown. Gene count is indicated by dot size and *P*(adjusted)‐value by colour. (E) Venn diagram depicting overlap of genes significantly regulated by ectopic FUS‐DDIT3‐EGFP expression (adjusted *P*‐value ≤ 0.05 and Log2 fold change≥ 1) and unique genes bound by FUS‐DDIT3 and at least one SWI/SNF component. Significant enrichment was determined by Fisher’s exact test (*P* < 1e‐10). (F) Venn diagram depicting overlap of BAF155, BRG1 and FUS‐DDIT3 ChIP binding sites in MLS 402‐91 from our dataset combined with BRD4 binding sites from Chen et al. dataset.

To determine the genomic binding sites of FUS‐DDIT3 and SWI/SNF, we performed chromatin immunoprecipitation followed by DNA sequencing in MLS 402‐91 cells using antibodies against DDIT3, BRG1 and BAF155, respectively. Both FUS‐DDIT3 and BRG1 bound DNA with the peak at the transcription start site, while BAF155 bound DNA with peaks about 300 base pairs upstream and downstream of the transcription start site, which roughly corresponds to the DNA bound by two nucleosomes (Fig. 5A). As the functional role of SWI/SNF complexes involves binding, moving and evicting nucleosomes, we speculate that the diverging profiles is due to epitope masking during ChIP or that inner core complexes with BAF155 but without the ATPase module bind chromatin in slightly different locations than fully assembled complexes. FUS‐DDIT3 and BRG1 displayed similar genomic binding features, while BAF155 bound somewhat more to promoters close to transcription start sites and less to distal intergenic DNA regions (Fig. 5B). Interestingly, around 8200 FUS‐DDIT3 binding sites annotated to 4461 unique genes overlapped with binding of at least one of the two SWI/SNF components (Fig. 5C). Enrichment analysis of the FUS‐DDIT3‐ and SWI/SNF‐bound genes with Gene ontology, Reactome and Hallmarks gene sets showed enrichment in gene expression processes, differentiation, locomotion and epithelial mesenchymal transition (Fig. 5D and Fig. S5A). To evaluate if binding by FET‐FOPs and SWI/SNF affected gene expression levels, we compared the ChIP data with RNA sequencing expression levels in HT1080 cells with ectopic FUS‐DDIT3 expression; 30% of genes with significantly changed mRNA expression by FUS‐DDIT3 expression (*n* = 810) overlapped with genes bound by FUS‐DDIT3 and at least one SWI/SNF component (significant enrichment, Fig. 5E). These genes had increased overlap with EZH2‐regulated genes, the enzymatic component of the PRC2 complex, as revealed by enrichment analysis with the Chemical and Genetic perturbation gene set (Fig. S5B).

To evaluate the genomic binding pattern of BRD4 in relation to FUS‐DDIT3 and SWI/SNF, we combined our dataset with the FUS‐DDIT3 and BRD4 ChIP‐seq datasets generated by Chen et al. in MLS 402‐91 cells [18]. In the latter, FUS‐DDIT3 and BRD4 bound DNA with the peak at the transcription start site even though BRD4 bound more often to promotors than FUS‐DDIT3 (Fig S5C and D). Combining the binding sites for FUS‐DDIT3, BRG1, BAF155 and BRD4 revealed that 45% of BRD4 and FUS‐DDIT3 overlapping binding sites also overlapped with at least one of the two SWI/SNF components (Fig. 5F), indicating that the SWI/SNF complex is involved in the reported BRD4‐dependent FUS‐DDIT3 function in MLS [18].

### 3.9 FET‐FOP interactomes are enriched in phase separation propensity and transcriptional components

**Fig. 6:**
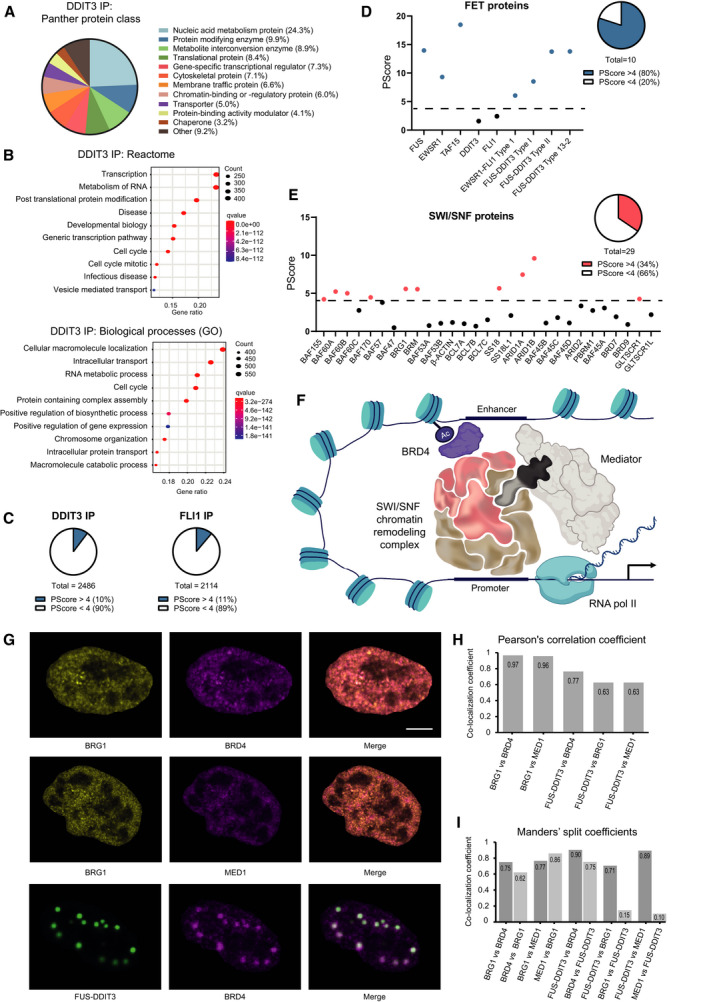
The interactomes of FET oncoproteins are enriched in phase separation propensity and transcriptional components. (A) Significantly enriched Panther protein class for FUS‐DDIT3‐interacting proteins. Percentage of proteins in protein class versus total of proteins matched to a protein class. (B) Significantly enriched gene sets from the “Reactome” and “Gene ontology (GO) biological processes” gene set collections for FUS‐DDIT3‐interacting proteins. Top 10 based on gene ratio is shown. Gene count is indicated by dot size and q‐value by colour. (C) Pie charts of FUS‐DDIT3 and EWSR1‐FLI1‐interacting proteins with phase separation propensity score (PScore) above or below the cutoff at 4. (D) Visualization of phase separation propensity score (PScore) of FET oncoproteins and their parental proteins. Pie chart shows proteins above or below the cutoff at 4. Blue dot indicates proteins with a high phase separation propensity, above the cutoff, visualized by black line. (E) Visualization of phase separation propensity score (PScore) of SWI/SNF components. Pie chart shows proteins above or below the cutoff at 4. Red dot indicates proteins with a high phase separation propensity, above the cutoff, visualized by black line. (F) Schematic visualization of potential BRD4, mediator, RNA polymerase II and FET‐FOP‐bound SWI/SNF complex interactions near chromatin. (G) Immunofluorescence staining of HT1080 cells transiently transfected with FUS‐DDIT3‐EGFP, probed with BRG1/BRD4, BRG1/MED1 and FUS‐DDIT3‐EGFP/BRD4 and analyzed with laser scanning microscopy. Representative images are shown. Scale bar: 5 µm. (H) Pearson’s correlation coefficient for co‐localization of BRG1 vs. BRD4, BRG1 vs. MED1, FUS‐DDIT3 vs. BRD4, FUS‐DDIT3 vs. BRG1 and FUS‐DDIT3 vs. MED1 in HT1080 cells transiently transfected with FUS‐DDIT3‐EGFP. (I) Manders’ split coefficients for co‐localization of BRG1 vs. BRD4, BRG1 vs. MED1, FUS‐DDIT3 vs. BRD4, FUS‐DDIT3 vs. BRG1 and FUS‐DDIT3 vs. MED1 in HT1080 cells transiently transfected with FUS‐DDIT3‐EGFP. The dark grey bar corresponds to the fraction of protein 1 (e.g. BRG1) overlapping with protein 2 (e.g. BRD4) and the light grey corresponds to the fraction of protein 2 (e.g. BRD4) overlapping with protein 1 (e.g. BRG1).

To learn more about the oncogenic mechanism involving FET oncoproteins, SWI/SNF complexes and BRD4, we further analyzed the proteins identified by mass spectrometry after DDIT3 and FLI1 IP. The interactomes of these FET‐FOPs were evaluated by enrichment analysis of Panther protein class, and Gene ontology, Reactome and Hallmarks gene sets (Fig. 6A‐B and Fig. S6A‐C). The enrichments were very similar for FUS‐DDIT3 and EWSR1‐FLI1 due to the many shared interaction partners. Enrichment analysis revealed substantial enrichment in transcription processes, RNA metabolism, cell cycle and protein modification processes, as well as MYC targets (Fig. 6A‐B and S6A‐C). This is supported by a previous study of the FUS‐DDIT3 interactome that showed a substantial enrichment of RNA processing proteins [48].

Around 1.2% of human proteins have a prion‐like domain, and therefore phase separation capacity, as evaluated by the prion‐like amino acid composition (PLAAC) algorithm [49, 50]. We used a phase separation predictor developed by Vernon et al., to evaluate the propensity of the FET‐FOPs and their interaction partners to form biomolecular condensates through phase separation [38]. Around 10% of the proteins pulled down by FET‐FOPs FUS‐DDIT3 and EWSR1‐FLI1 displayed a PScore above the cutoff at 4 indicating enrichment of proteins with phase separation properties (Fig. 6C). FET proteins, via their prion‐like N‐terminal domain, are known to form biomolecular condensates [51]. Indeed, all of them had a high PScore: 14.0, 9.3 and 18.5, respectively (Fig. 6D). While the transcription‐factor partners DDIT3 and FLI1 had low PScores (1.6 and 2.4, respectively), the FET‐FOPs maintained the majority of the phase separation propensity from the FET proteins (FUS‐DDIT3 type II/5‐2: 8.5, type I/7‐2 and type 13‐2: 13.8, and EWSR1‐FLI1: 6.0). Furthermore, the addition of exon 6 and 7 of FUS increased the predicted phase separation potential of the fusion oncoprotein substantially (Fig. 6D). Interestingly, SWI/SNF proteins also had enriched phase separation capacity with 34% of SWI/SNF components above the PScore cutoff (BRG1, BRM, BAF155, BAF170, SS18, ARID1A‐B, BAF60A‐B and GLTSCR1) (Fig. 6E). These proteins almost completely overlapped with the SWI/SNF components described by Davis et al., to have prion‐like domains based on the PLAAC algorithm [52].

Further inspection of the FET‐FOP IP‐MS data revealed that BRD4 and SWI/SNF components were accompanied by many subunits of the mediator complex as well as RNA polymerase II suggesting that part of the transcriptional machinery reside together with FET‐FOPs, SWI/SNF and BRD4 near chromatin (Table S3 and Fig. 6F). Immunofluorescence analysis of HT1080 cells transiently transfected with FUS‐DDIT3‐EGFP and stained for BRG1, BRD4 and the MED1 mediator component showed overlapping localization between all tested proteins (Fig 6G‐I and Fig. S6D). The BRG1/BRD4 and BRG1/MED1 stains overlapped extensively whereas the FUS‐DDIT3‐EGFP signal overlapped only partially with BRD4, BRG1 and MED1. This is mainly because FUS‐DDIT3 was strongly enriched in nuclear puncta, as previously described by us [27, 53], while SWI/SNF components, BRD4 and the mediator complex showed a more disperse localization over the nuclei. Most of the FUS‐DDIT3‐EGFP signal thus overlapped with the tested proteins while BRG1, BRD4 and MED1 were present also in parts not occupied by the FUS‐DDIT3 protein. Moreover, in cells with FUS‐DDIT3‐EGFP nuclear puncta formation, BRD4 but not BRG1 were enriched in the nuclear structures (Fig. 6G and Fig. S6D).

## 4 Discussion

FET fusion oncoproteins, guided by their transcription‐factor partner, bind to specific genomic binding sites and induce oncogenic transcriptional profiles in FET‐FOP‐caused cancers [54, 55]. The transcriptional coactivator BRD4 and SWI/SNF chromatin remodelling complexes are involved in transcriptional regulation. Earlier studies have shown that normal and oncogenic FET proteins bind to the SWI/SNF chromatin remodelling complex via their shared low complexity N‐terminal parts [2, 3]. In this study, we characterize the interactions between FET oncoproteins FUS‐DDIT3 and EWSR1‐FLI1, the SWI/SNF chromatin remodelling complexes and BRD4. We show that FET oncoproteins bind to all three main SWI/SNF subtypes cBAF, PBAF and the recently characterized GBAF/ncBAF. The different subtypes consist of both shared and subtype‐specific protein components, each contributing to the overall functions of the complexes. The binding of FET‐FOPs may thus affect the diverse functions of all three main SWI/SNF subtypes. FET oncoproteins have been reported to recruit SWI/SNF complexes to novel genomic loci [3] but there are no other effects on SWI/SNF functions described. We show that the core complexes, present in all three SWI/SNF subtypes, remain intact when FET‐FOPs are bound but cannot completely rule out that one or more subtype‐specific components are lost. Interestingly, we observed a preferential immunoprecipitation of FET‐FOP‐bound SWI/SNF complexes that may be explained by binding to a hitherto undefined SWI/SNF subgroup, or that binding of FET‐FOPs have an effect on SWI/SNF complex conformation.

The direct interaction partner(s) of FET‐FOPs in the SWI/SNF complex has not yet been defined. In a recent study of Ewing sarcoma cells, Selvanathan et al. reported that FET‐FOPs bind directly to ARID1A, a cBAF‐specific component [56]. This conclusion was made based on recombinant protein interaction studies. Furthermore, knockdown of ARID1A completely disrupted co‐IP of SWI/SNF components with EWSR1‐FLI1. However, this conclusion is not compatible with our results showing that the FET‐FOPs FUS‐DDIT3 and EWSR1‐FLI1 bind all three SWI/SNF subtypes, including those lacking ARID1A. A possible explanation for this discrepancy is that ARID1A knockdown disrupts the SWI/SNF complex and surrounding subunits common to all SWI/SNF subtypes so that the interaction is lost. Our results are compatible with FET‐FOPs binding to all the related and mutually exclusive subunits ARID1A/ARID2 and corresponding GBAF‐subunit GLTSCR1/1L, and/or to a core SWI/SNF component. A very recent report supports this conclusion; Davis et al. proposed that FET‐FOPs interact with SWI/SNF via the prion‐like domain in the FET N‐terminal domain and prion‐like domains present in several SWI/SNF components including BRG1, ARID1A/B, and BAF155/BAF170 [52].

The SWI/SNF core complex is reportedly very robust and remains intact up to about 2.5 m urea [57]. Here, we used sequential salt extraction analysis followed by IP to study SWI/SNF and FET protein interactions. Although normal FET proteins bound stronger to chromatin compared to FET‐FOPs and were extracted at larger amounts in the 1000 mm salt fraction, only a small amount bound to SWI/SNF in this fraction. These results are in agreement with the reported diverse functions and binding partners of normal FET proteins [58, 59]. In contrast, all FET‐FOPs extracted in the 1000 mm salt fraction were bound to SWI/SNF, indicating strong interactions. Our results partially differ from other studies reporting a transient interaction of the SWI/SNF complex with the EWSR1‐FLI1 fusion oncoprotein in Ewing sarcoma [3] and TMPRSS2‐ERG in prostate cancer [60]. This discrepancy may be explained by different conditions during extraction and downstream analysis; we intentionally avoided the use of detergents during nuclear extraction and during IP in order not to diminish protein interactions.

The bromodomain protein BRD4 that recognizes and binds to acetylated histone tails, e.g. in super‐enhancers, has important roles in transcription and splicing control. Moreover, BRD4 inhibition has gained interest, in several tumour types, as a novel strategy to target oncogenic transcription factors aberrantly activated by super‐enhancers [61]. Using the dual‐bromodomain inhibitor AZD5153, we show that FET sarcoma cells are sensitive to BRD4 inhibition. The sensitivity of these tumour cells to BRD4 inhibition was recently attributed to a physical interaction between BRD4 and FET‐FOPs in MLS [18] and EWS [19]. However, our experiments show that both FET‐FOPs and BRD4 bind the SWI/SNF complex and that the amount of BRD4 that co‐immunoprecipitates with SWI/SNF do not change after forced expression of FET oncoproteins. In addition, immunofluorescence analysis showed that BRD4 and BRG1 co‐localize substantially. Our results thus suggest that the BRD4 interaction is mediated by the SWI/SNF complex. Furthermore, our results show that the binding between FET‐FOPs and SWI/SNF was not disrupted by BRD4 bromodomain inhibition. Inhibition of BRD4 binding to acetylated chromatin regions such as super‐enhancers is therefore the most plausible mechanism behind the effect of BRD4 bromodomain inhibition on FET sarcoma cells.

Our ChIP‐seq analysis showed that SWI/SNF components, FET‐FOPs and BRD4 co‐localize on chromatin and regulate a set of target genes. In addition, our IP‐MS data suggest that they reside together in large protein complexes that also interact with subunits of the mediator complex and RNA polymerase II. Indeed, immunofluorescence analysis showed that BRG1 co‐localizes substantially with MED1. In support of this, EWSR1‐FLI1 has been found in a large transcriptional complex with BRD4, MED1 and RNA polymerase II [19]. Taken together, these results may provide a possible mechanism behind transcriptional deregulation and FET‐FOP‐induced expression profiles. In support of this, in Ewing sarcoma, the EWSR1‐FLI1‐induced *de novo* super‐enhancers and oncogenic transcriptional programmes [41, 62] were disrupted and repressed by BRD4 inhibition or depletion [63, 64, 65]. Likewise in myxoid liposarcoma, FUS‐DDIT3 and BRD4 were reported to co‐localize on enhancers and regulate expression of super‐enhancer‐associated genes [18]. Almost 20 years ago, we showed that FET‐FOPs form subnuclear aggregates [27]. The nature of these nuclear structures can be explained by recent studies showing that FET oncoproteins form biomolecular condensates at binding‐specific loci through liquid‐liquid phase separation where they recruit RNA polymerase II and activate transcription [52, 66, 67, 68]. We used a phase separation predictor developed by Vernon et al. [38] and show that FET‐FOPs and SWI/SNF components are enriched in phase separation capacity. Since amino acid sequences with high propensity for pi interactions are more likely to self‐associate, proteins with high PScores would be more likely to interact with each other [38]. Indeed, we showed that both FUS‐DDIT3 and EWSR1‐FLI1 interactomes were enriched in phase separation capacity. The phase separation capacity of FET‐FOPs, via the low complexity FET N‐terminal domain, is shared by coactivators, such as BRD4 and mediator, which are suggested to form transcriptional hubs at super‐enhancers [69]. This applies to both the short and long BRD4 isoform [70]. Furthermore, phase separation processes, although controversial, may explain why FET sarcoma cells are sensitive to BRD4 inhibition; a recent study suggested that the preferential disruption of super‐enhancer‐driven gene expression by BRD4 inhibitors can be attributed to a preferential partitioning of these drugs into phase‐separated condensates at super‐enhancers [71]. The findings above together with our results showing co‐precipitation and genomic co‐localization of these proteins and complexes, suggest a wide interference of FET‐FOPs in genomic programming and may explain the enigmatic gene expression profiles of FET‐FOP‐caused tumours.

Interestingly, the possible oncogenic role of the BRD4–SWI/SNF interaction extends to far more cancer types than those caused by FET oncoproteins. BRD4 is also a drug target in cancers with aberrant SWI/SNF function, caused by mutated SWI/SNF subunits BRG1 or BRM [72]. Furthermore, in another fusion‐driven tumour form, rhabdomyosarcoma, the PAX3‐FOXO1 fusion oncoprotein was shown to interact with BRD4 and induce oncogenic transcription [73] in a similar way as EWSR1‐FLI1. In prostate cancer, the TMPRSS2‐ERG fusion protein was also shown to interact with BRD4 [74] and with SWI/SNF [60]. Our finding that the interaction between FET‐FOPs and BRD4 is mediated by the SWI/SNF complex, suggests that the interactions between other fusion oncoproteins and BRD4, and at least part of the oncogenic effect, could be mediated via the SWI/SNF chromatin remodelling complex also in those tumour types.

## 5 Conclusions

In this study, we studied the FET oncoproteins FUS‐DDIT3 and EWSR1‐FLI1 and show that they bind to all three SWI/SNF subtypes cBAF, PBAF and GBAF. Our results indicate that the interaction between FET oncoproteins and BRD4 is mediated by the common interactions with the SWI/SNF chromatin remodelling complex. The BRD4–SWI/SNF–FET‐FOP complexes are very robust, withstanding 1000 mm salt. In addition, sequential salt extraction analysis shows that normal FET proteins bind stronger to chromatin but have a substantially weaker interaction with SWI/SNF than oncogenic FET proteins. BRD4, FET‐FOPs and SWI/SNF co‐localize on chromatin and reside in complexes that most likely interact with mediator and RNA Polymerase II. Further studies are needed to elucidate how FET oncoproteins, SWI/SNF complexes and BRD4 establish an aberrant epigenetic landscape including *de novo* enhancers, and their impact on chromatin remodelling activities and downstream oncogenic transcriptional output. In addition, it remains to be elucidated whether all FET‐FOPs share the same properties as FUS‐DDIT3 and EWSR1‐FLI1 or if their transcription‐factor parts mediate distinct functions and interaction partners such as diverse SWI/SNF subtypes. We conclude that BRD4 and SWI/SNF chromatin remodelling complexes emerge as interesting future drug targets in FET‐FOP‐caused cancers as well as in other cancer types with aberrant SWI/SNF function.

## Conflict of interest

AS is a board member and declares stock ownership in Tulebovaasta, Iscaff Pharma and SiMSen Diagnostics.

### Peer Review

The peer review history for this article is available at https://publons.com/publon/10.1002/1878‐0261.13195.

## Author contributions

ML designed the study, performed the main laboratory work, planned and designed experiments, analyzed the data, prepared the figures and wrote the manuscript. CV performed the cell viability assays and analysis. TÖ performed ChIP‐seq and PScore analysis. LA performed the RNA‐seq and ChIP‐seq analysis. AO performed ChIP‐seq experiments and analysis. ME helped with cell culture and nuclear sample preparations, and performed the BRD4 degrader test. HF and AS provided strategic and scientific input. PÅ designed the study, contributed with data interpretation and wrote the manuscript. All authors revised the manuscript, read and approved the final manuscript.

## Supporting information


**Fig. S1**. Expression levels of SWI/SNF components and FET‐FOPs in sarcoma cells.

**Fig. S2**. Detailed binding profiles from sequential salt extracts.

**Fig. S3**. Quantitative western blot.

**Fig. S4**. BRD4 expression, degradation and co‐immunoprecipitation.

**Fig. S5**. ChIP‐seq and enrichment analysis.

**Fig. S6**. Enrichment analysis of FET oncoprotein interactomes and immunofluorescence analysis.

**Table S1**. Primary antibodies used for western blot analysis.

**Table S2**. Total protein amount in sequential salt fractions.

**Table S3**. MS‐identified SWI/SNF, mediator and RNA polymerase II components after FET‐FOP IP.

**Data S1**. Full Western blot membranes.

**Data S2**. Gene expression array of SWI/SNF components (Fig. 1D).

**Data S3**. RNA sequencing: differential expression of SWI/SNF components (Fig. S1C‐D).
**Data S4**. Quantification western blot sequential salt extraction signals (Fig. 2 and Fig. S2).
**Data S5**. Cell viability assay AZD5153 and ARV‐825 (Fig. 4F and Fig. S4F).
**Data S6**. ChIP‐annotated genes overlap (Fig. 5).
**Data S7**. ChIP: Gene set enrichment analysis (Fig. 5D and Fig. S5A‐B).
**Data S8**. ChIP/RNA‐seq: Gene overlap (Fig. 5E).
**Data S9**. MS: Enrichment analysis (Fig. 6A‐B and Fig. S6).
**Data S10**. FET‐FOP interactome and Pscore (Fig. 6C‐E).

## Data Availability

Full Western blot membranes and source data for gene expression arrays, RNA sequencing, quantifications of WB signals, cell viability assays, ChIP‐seq and MS data generated during the current study are available as supporting information. Raw and normalized microarray data generated during the current study were deposited according to MIAME standards in NCBI's Gene Expression Omnibus with GEO Series accession number GSE167270 [https://www.ncbi.nlm.nih.gov/geo/query/acc.cgi?acc=GSE167270]. The ChIP‐seq data (raw fastq‐files) generated during the current study were deposited to the NCBI Sequence Read Archive (SRA) with accession number PRJNA721591 [https://www.ncbi.nlm.nih.gov/sra]. Publicly available datasets used in this study are described in each method section.
