# Supplementary material for: FET fusion oncoproteins interact with BRD4 and SWI/SNF chromatin remodelling complex subtypes in sarcoma
Source: Mol Oncol. 2022 Mar 19;16(13):2470–95. doi: 10.1002/1878-0261.13195 (PMC9251840; doi:10.1002/1878-0261.13195)
Supplement: Supplementary file 1 — Fig. S1. Expression levels of SWI/SNF components and FET‐FOPs in sarcoma cells. [file MOL2-16-2470-s007.pdf]

Supporting information file 1. Supplementary Figures and Tables

FET fusion oncoproteins interact with BRD4 and SWI/SNF chromatin remodelling complex subtypes in sarcoma

Malin Lindén[1], Christoffer Vannas[1], Tobias Österlund[1,2], Lisa Andersson[1], Ayman Osman[1], Mandy Escobar[1], Henrik Fagman[1], Anders Ståhlberg[1,2,3*] and Pierre Åman[1*]

[1]Sahlgrenska Center for Cancer Research, Institute of Biomedicine, Department of Laboratory Medicine, Sahlgrenska Academy, University of Gothenburg, Box 425, 40530, Gothenburg, Sweden

[2]Wallenberg Centre for Molecular and Translational Medicine, University of Gothenburg, Sweden

[3]Region Västra Götaland, Sahlgrenska University Hospital, Department of Clinical Genetics and Genomics, Gothenburg, Sweden

*Corresponding authors**:** Prof. Pierre Åman: pierre.aman@gu.se; and Ass. Prof. Anders Ståhlberg: anders.stahlberg@gu.se

Table of Contents

Supporting information file 1. Supplementary Figures and Tables 1
Supplementary Figure 1 2
Supplementary Figure 2 4
Supplementary Figure 3 6
Supplementary Figure 4 7
Supplementary Figure 5 9
Supplementary Figure 6 10
Supplementary Table 1 11
Supplementary Table 2 12
Supplementary Table 3 12

Supplementary Figure 1

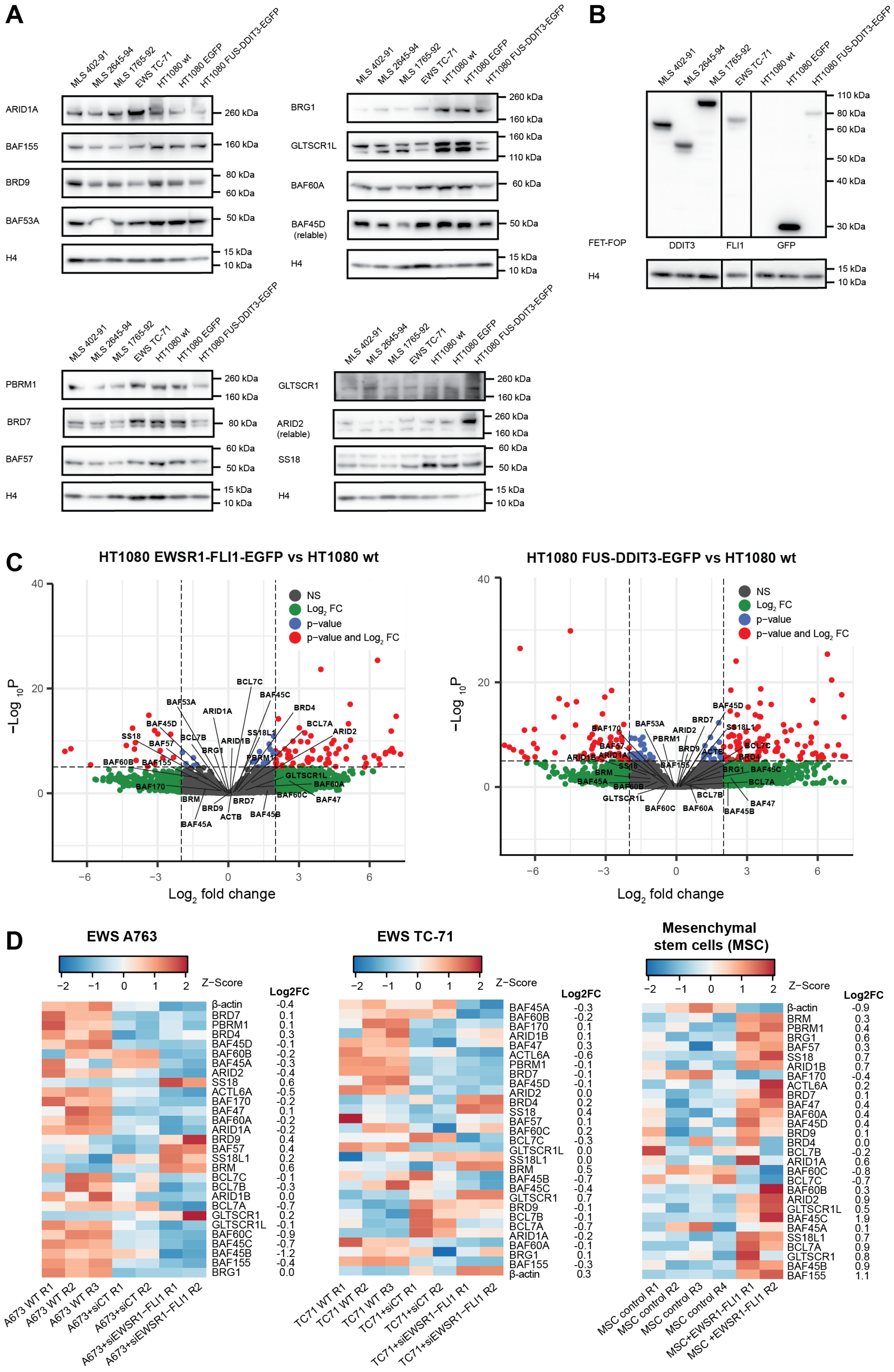

Supplementary Fig. 1. Expression levels of SWI/SNF components and FET-FOPs in sarcoma cells.

A. Western blot (WB) from Fig. 1c with antibodies grouped according to original membrane and histone H4 loading control for each membrane; 10 µg nuclear extracts (extracted in 500 mM KCl) visualizing SWI/SNF components in myxoid liposarcoma (MLS 402-91, 2645-94 and 1765-92), Ewing sarcoma (EWS TC-71) and HT1080 fibrosarcoma (wt, EGFP or FUS-DDIT3-EGFP) cells using antibodies against ARID1A, BAF155, BRD9, BAF53A, BRG1, GLTSCR1L, EZH2, BAF60A, BAF45D, PBRM1, BRD7, BAF57, GLTSCR1, ARID2 and SS18 respectively, with histone H4 as loading control. Relabeled (stripped and reprobed) membrane parts are indicated.

B. Western blot of 10 µg nuclear extracts (extracted in 500 mM KCl) visualizing FET oncoproteins in myxoid liposarcoma (MLS 402-91, 2645-94 and 1765-92), Ewing sarcoma (EWS TC-71) and HT1080 fibrosarcoma (wt, EGFP or FUS-DDIT3-EGFP) cells using antibodies against DDIT3, FLI1 and GFP respectively, with histone H4 as loading control. Note that the MLS cell lines contain different variants of FUS-DDIT3 (7-2: type I, 5-2: type II and 13-2, respectively) and are thus detected at different sizes.

C. Volcano plots of RNA sequencing data show differentially expressed genes between HT1080 EWSR1-FLI1-EGFP or HT1080 FUS-DDIT3-EGFP and HT1080 wt as control. Black vertical lines highlight log fold changes (Log_2 FC) of ±2, while the black horizontal line represents a p-value of 1e-5. Colored dots indicate individual genes with gene expression differences that were significant (above the cutoff) based on both p-value and fold change (red), only fold change (green), only p-value (blue) or not significant (NS) in either (grey). SWI/SNF components are highlighted; the majority had no significant expression differences.

D. Clustered heat maps of publicly available RNA sequencing data visualizing expression levels for SWI/SNF components in EWS A763 (wt: n=3, siCT control siRNA: n=2, siEWSR1-FLI1: n=2), EWS TC-71 (wt: n=3, siCT control siRNA: n=2, siEWSR1-FLI1: n=2), and in mesenchymal stem cells (MSC control, n=4) with ectopic expression of EWSR1-FLI1 (n=2). Log2FC= Log2 Fold change of siEWSR1-FLI1 versus siCT (left and middle) or ectopic expression versus control (right) is shown.

Supplementary Figure 2

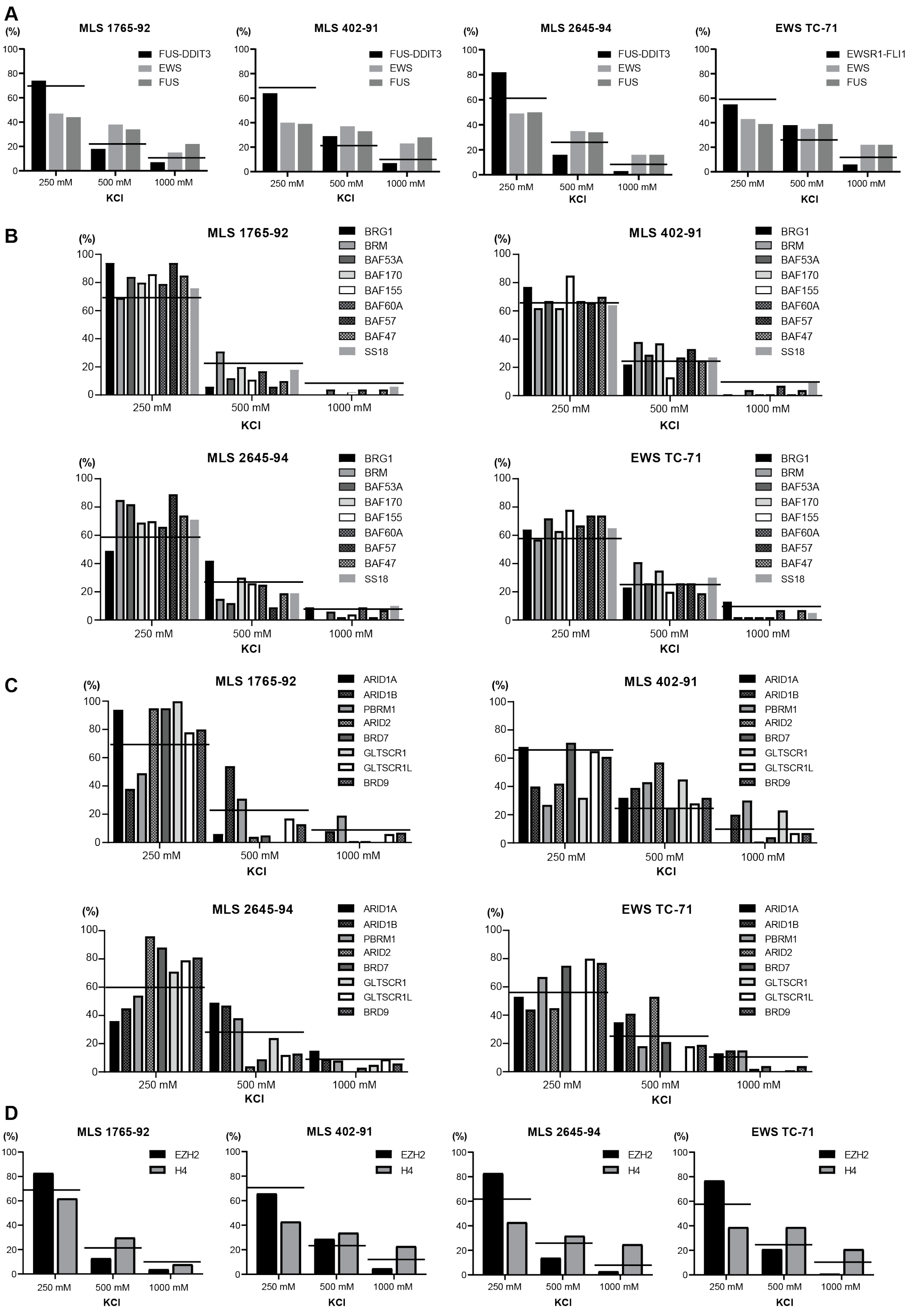

Supplementary Fig. 2. Detailed binding profiles from sequential salt extracts.

A. Bar chart visualizing percentage of FET-FOPs (FUS-DDIT3 or EWSR1-FLI1), EWSR1 and FUS extracted in each salt fraction (250, 500 and 1000 mM) for MLS 1765-92, MLS 402-91, MLS 2645-94 and EWS TC-71, respectively.

B. Bar chart visualizing percentage of SWI/SNF core components BRG1, BRM, BAF53A, BAF170, BAF155, BAF60A, BAF57, BAF47 and SS18 extracted in each salt fraction (250, 500 and 1000 mM) for MLS 1765-92, 402-91 and 2645-94, and EWS TC-71, respectively.

C. Bar chart visualizing percentage of SWI/SNF-subtype-specific subunits: ARID1A and ARID1B (cBAF), PBRM1, ARID2 and BRD7 (PBAF), and GLTSCR1, GLTSCR1L and BRD9 (GBAF/ncBAF) extracted in each salt fraction (250, 500 and 1000 mM) for MLS 1765-92, 402-91 and, 2645-94 and EWS TC-71, respectively.

D. Bar chart visualizing percentage of the PRC2-component EZH2 and Histone H4 control extracted in each salt fraction (250, 500 and 1000 mM) for MLS 1765-92, 402-91 and 2645-94 and EWS TC-71, respectively.

Graphs are based on western blot signals quantified from Figure 2. Black lines indicate percentage of total protein extracted in each fraction during sequential salt extraction for each cell line (see Supplementary Table 2).

Supplementary Figure 3

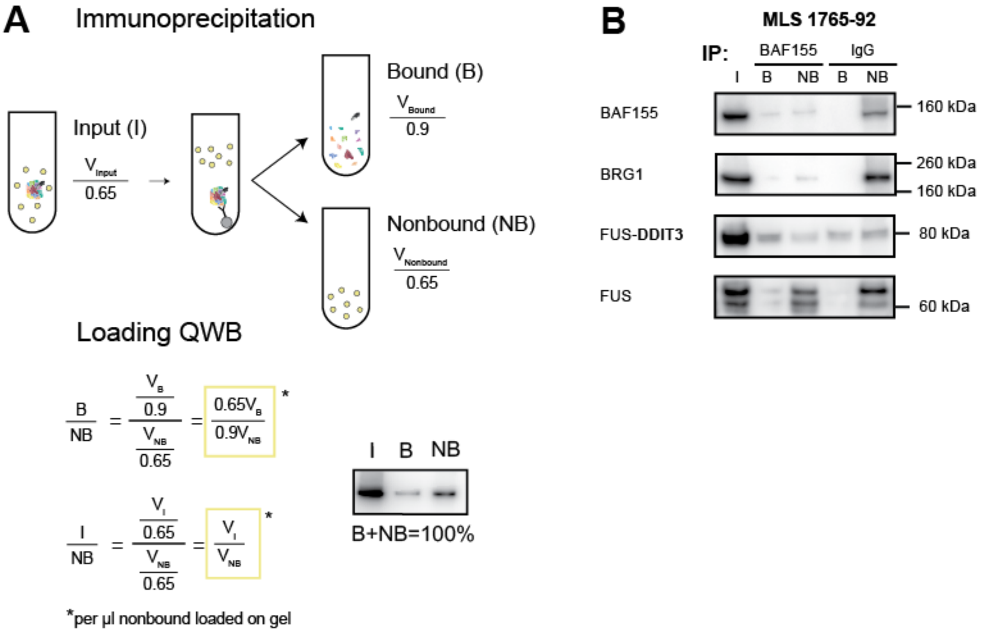

Supplementary Fig. 3. Quantitative western blot.

A. Quantitative Western blot (QWB) procedure: During Immunoprecipitation (IP), nuclear proteins end up either in the bound (B) or nonbound (NB) fraction. Before QWB loading, input (I) samples and eluate (bound, B) samples are diluted relative NB, by taking into account dilutions during IP such as the different volumes (V) and WB-dilution factors (0.65 or 0.9: Input and nonbound are “diluted” in 25% LDS sample buffer and 10% sample reducing agent before WB while eluates are only diluted in 10% sample reducing agent). The detected signals can then be compared directly, because B+NB now equals 100%.

B. Quantitative Western blot analysis of BAF155-biotin immunoprecipitated nuclear extracts of MLS 1765-92 cell line, visualizing SWI/SNF components (BAF155 and BRG1), FET-FOP FUS-DDIT3 (ab against C-terminal partner) and normal FET protein FUS. Input (I) and eluate (bound, B) samples were diluted relative nonbound (NB), so that B+NB=100%. Around 5% of input was loaded on the gel. Another replicate is shown in Fig. 3b.

Supplementary Figure 4

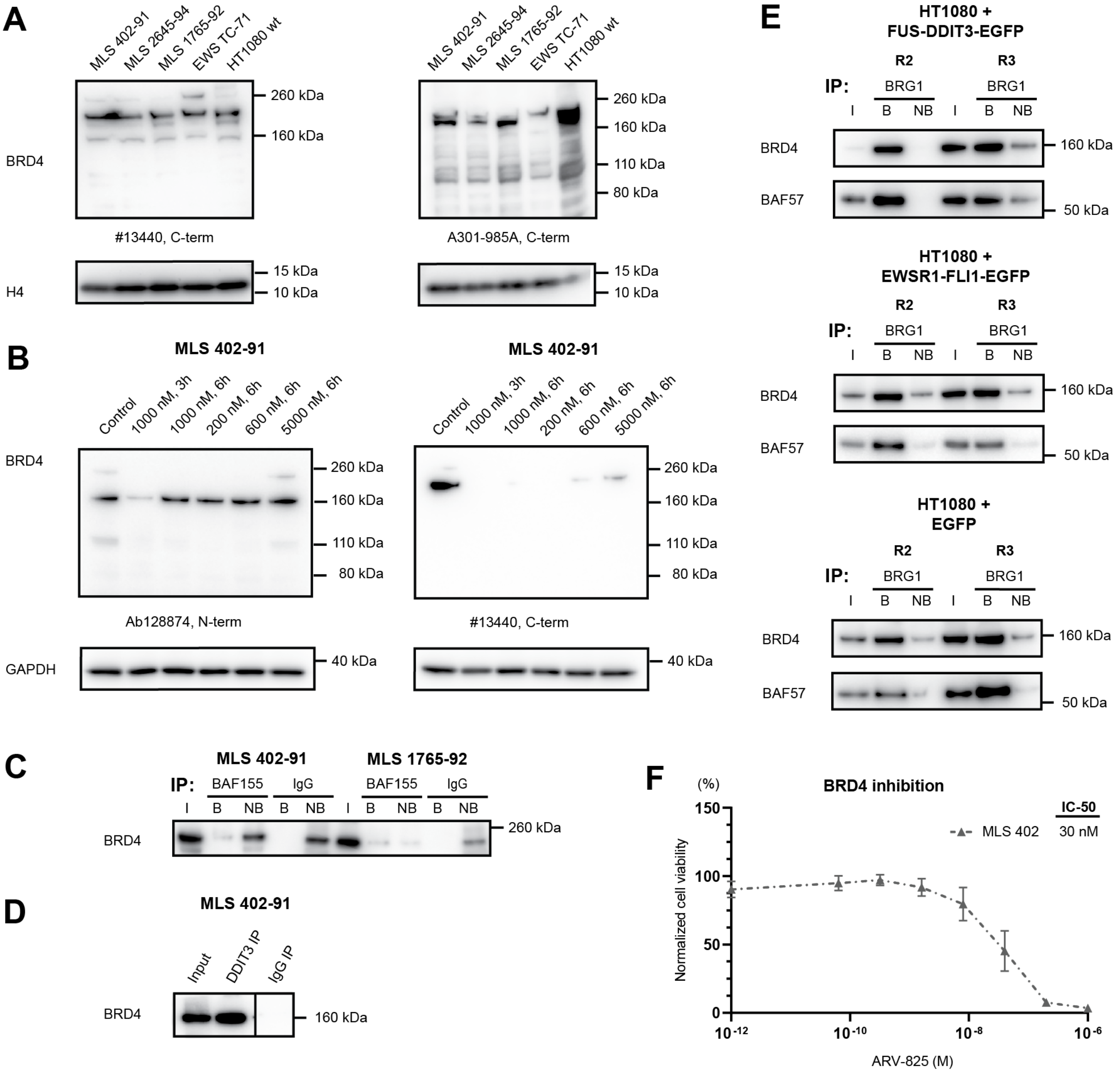

Supplementary Fig. 4. BRD4 expression, degradation and co-immunoprecipitation.

A. Western blot analysis of BRD4 isoforms using 10 µg nuclear extracts (extracted in 500 mM KCl) of MLS 402-91, 2645-94 and 1765-92, EWS TC-71 and HT1080 fibrosarcoma cell line, using two BRD4 antibodies: #13440 (C-term, long isoform) and A301-985A (C-term, long isoform). Note that they specifically detect the larger potentially post-translational-modified 200 kDa version of the BRD4 long isoform. Another BRD4 antibody, targeting the N-terminus, is shown in Fig. 4b.

B. Western blot analysis of BRD4 isoforms using 8 µg whole-cell extracts after treatment with BRD4 degrader ARV-825 (control, 1000 nM for 3h, 1000 nM for 6h, 200 nM for 6h, 600 nM for 6h or 5000 nM for 6h) visualized by two BRD4 antibodies: ab128874 (N-term, both short and long BRD4 isoform) and #13440 (C-term, long isoform). Note that the drug has a known loss of efficacy at too high drug concentrations due to dimerization.

C. Quantitative western blot analysis of BAF155-biotin immunoprecipitated (IP) nuclear extracts of MLS 402-91 and 1765-92 sarcoma cell lines, visualizing co-IP of BRD4 (antibody #13440). Same samples and loading as in Fig. 3b.

D. Western blot analysis of DDIT3-biotin immunoprecipitated nuclear extracts of MLS 402-91, visualizing successful co-IP of BRD4 (antibody ab128874). Same samples and loading as in Fig. 1e.

E. Western blot analysis of BRG1-biotin immunoprecipitated nuclear extracts of HT1080 fibrosarcoma cells transiently transfected (24h) with FUS-DDIT3-EGFP, EWSR1-FLI1-EGFP or EGFP control (biological replicates R2-R3) visualizing successful co-IP of the SWI/SNF complex (BAF57) and BRD4. Maximum amount of eluate (B) and nonbound (NB) were loaded on the gel. Input (I) samples were diluted relative NB and around 5% of input was loaded. Replicate R1 is shown in Fig. 4e.

F. Cell viability dose response curve of MLS cell line 402-91 after BRD4 degradation (BRD4 inhibition, ARV-825, 72h) with IC-50 value displayed. Mean +/- SD (standard deviation) is shown, n = 12 (2 biological, 6 technical replicates each).

Supplementary Figure 5

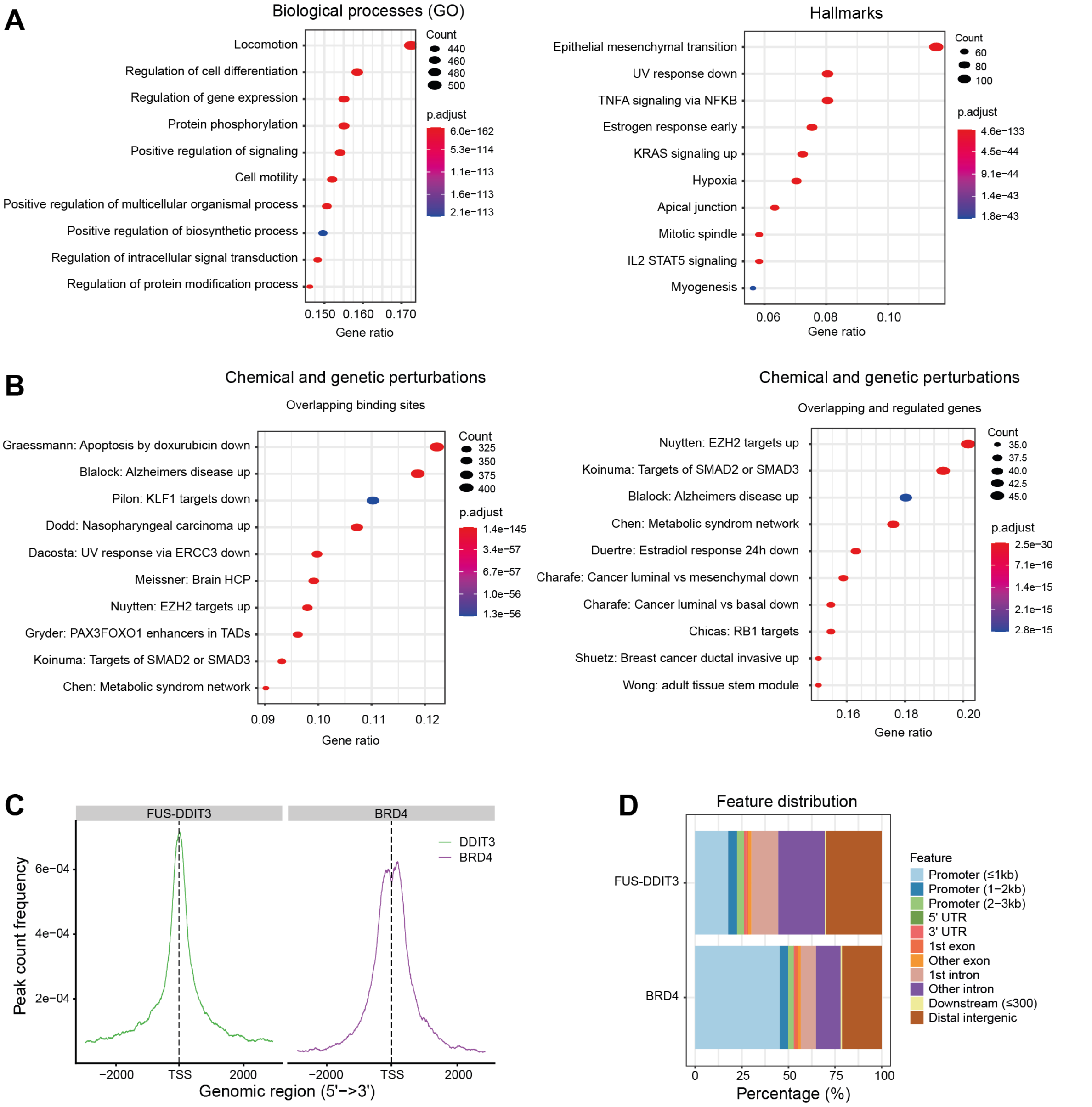

Supplementary Fig. 5. ChIP-seq and enrichment analysis.

A. Significantly enriched gene sets from “Gene ontology (GO) biological processes” and “Hallmarks” gene set collections using the 4461 unique genes bound by FUS-DDIT3 and at least one SWI/SNF component. Top 10 based on gene ratio is shown. Gene count is indicated by dot size and p(adjusted)-value by color.

B. Significantly enriched gene sets from “Chemical and genetic perturbation” gene set collection using the unique genes bound by FUS-DDIT3 and at least one SWI/SNF component (left, n=4461) and genes overlapping with significantly regulated genes (right, n=240). Top 10 based on gene ratio is shown. Gene count is indicated by dot size and p(adjusted)-value by color.

C. ChIP-seq peak profiles of FUS-DDIT3 and BRD4 in MLS 402-91 from Chen *et al.* dataset ± 3 kb surrounding transcription start site (TSS).

D. Bar chart showing the genomic distribution of FUS-DDIT3 and BRD4 ChIP-seq peaks in MLS 402-91 from Chen *et al.* dataset.

Supplementary Figure 6

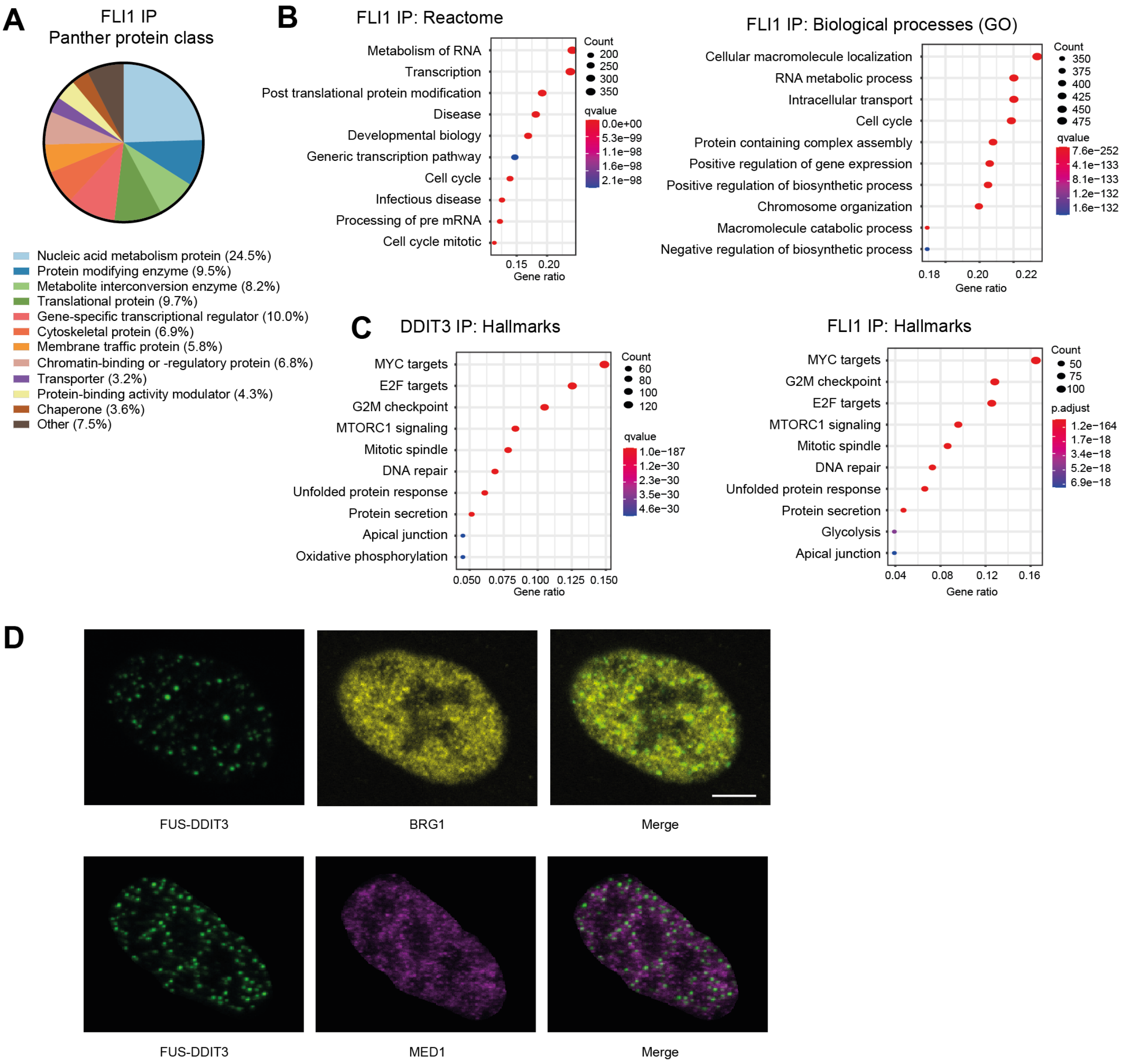

Supplementary Fig. 6. Enrichment analysis of FET oncoprotein interactomes and immunofluorescence analysis.

A. Significantly enriched Panther protein class for EWSR1-FLI1-interacting proteins. Percentage of proteins in protein class versus total of proteins matched to a protein class. Protein class ranked according to DDIT3 IP (Fig. 6a).

B. Significantly enriched gene sets from the “Reactome” and “Gene ontology (GO) biological processes” gene set collections for EWSR1-FLI1-interacting proteins. Top 10 based on gene ratio is shown. Gene count is indicated by dot size and q-value by color.

C. Significantly enriched gene sets from the “Hallmarks” gene set collection for FUS-DDIT3- and EWSR1-FLI1-interacting proteins. Top 10 based on gene ratio is shown. Gene count is indicated by dot size and q-value or adjusted p-value by color.

D. Immunofluorescence stainings of HT1080 cells transiently transfected with FUS-DDIT3-EGFP, probed with FUS-DDIT3-EGFP/BRG1 or FUS-DDIT3-EGFP/MED1 and analyzed with laser scanning microscopy. Representative images are shown. Scale bar: 5 µm.

Supplementary Table 1

Supplementary Table 1. Primary antibodies used for western blot analysis.

	Company	Product number	Amount	Block buffer	Species	Molecular weight/ Detected size
ARID1A	Atlas Antibodies	HPA005456	0.2 µg/ml	Milk	Rabbit	~250 kDa
ARID1B	Bethyl	A301-047A	0.5 µg/ml	Milk	Rabbit	~250 kDa
ARID2	Santa Cruz	sc-166117	1 µg/ml	BSA	Rabbit	~200 kDa
BAF45D	Abcam	Ab134942	0.8 µg/ml	Milk	Rabbit	~50 kDa
BAF47	Abcam	Ab12167	1.1 µg/ml	BSA	Rabbit	~45 kDa
BAF53A	Abcam	Ab131272	0.4 µg/ml	Milk/BSA	Rabbit	~50 kDa
BAF57	Abcam	Ab131328	0.04 µg/ml	Milk/BSA	Rabbit	~55 kDa
BAF60A	Santa Cruz	sc-135843	0.1 µg/ml	Milk	Mouse	~60 kDa
BAF155 (DXD7)	Santa Cruz	sc-32763	1.0 µg/ml	BSA	Mouse	~155 kDa
BAF170 (E-6)	Santa Cruz	sc-17838	0.5 µg/ml	BSA	Mouse	~170 kDa
BRD4 (C-term)	Cell signaling	13440	1:2000	Milk	Rabbit	~200[1] kDa
BRD4 (N-term)	Abcam	Ab128874	1 µg/ml	BSA	Rabbit	~160 kDa
BRD4 (C-term)	Bethyl	A301-985A	0.3 µg/ml	Milk	Rabbit	~200[1] kDa
BRD7 (B-8)	Santa Cruz	Sc-376180	1 µg/ml	BSA	Mouse	~75 kDa
BRD9	Abcam	Ab137245	0.5 µg/ml	BSA	Rabbit	~75 kDa
BRG1 (G-7)	Santa Cruz	sc-17796	0.2 µg/ml	BSA	Mouse	~190 kDa
BRM	Abcam	Ab15597	0.5 µg/ml	Milk	Rabbit	~190 kDa
DDIT3	Proteintech	15204-1	0.7 µg/ml	Milk	Rabbit	NA
DDIT3 (9C8)	Abcam	Ab11419	1 µg/ml	Milk	Mouse	NA
EWSR1 (G-5)	Santa Cruz	sc-28327	0.2 µg/ml	Milk	Mouse	~80 kDa
EZH2	Merck Millipore	#07-689	0.5 µg/ml	Milk	Rabbit	~90 kDa
FLI1	Abcam	Ab15289	0.2 µg/ml	Milk	Rabbit	NA
FUS (4H11)	Santa Cruz	sc-47711	0.2 µg/ml	Milk	Mouse	~60 kDa
GAPDH	Proteintech	60004-1-Ig	0.02 µg/ml	BSA	Mouse	~35 kDa
GFP	Clontech	632381	1 µg/ml	Milk	Mouse	NA
GLTSCR1 (H-10)	Santa Cruz	sc-515086	1 µg/ml	BSA	Rabbit	~200 kDa
GLTSCR1L	Atlas Antibodies	HPA029391[2]	0.5 µg/ml	Milk	Rabbit	~140 kDa
Histone H4	Merck Millipore	#04-858	1:30 000	Milk	Rabbit	~ 12 kDa
PBRM1	Bethyl	A301-591A	0.2 µg/ml	Milk	Rabbit	~200 kDa
SS18 (H80)	Santa Cruz	sc-28698	1 µg/ml	Milk	Rabbit	~50 kDa

[1] Post-translational modified version of the BRD4 long isoform (150 kDa), see Fig. S4a.

2 Same antibody as PA556126, Invitrogen

NA. Not applicable, varies depending on construct and fusion type.

Supplementary Table 2

Supplementary Table 2. Total protein amount in sequential salt fractions.

Fraction	MLS 1765-92		MLS 402-91		MLS 2645-94		EWS TC-71	
	(μg)	(%)	(μg)	(%)	(μg)	(%)	(μg)	(%)
**250 mM**	1195	68	1366	66	760	60	1204	59
**500 mM**	379	22	463	22	355	28	557	27
**1000 mM**	171	10	231	11	143	11	283	14

Total amount and percentage of proteins extracted in each fraction (250 mM, 500 mM, and 1000 mM KCl) during sequential salt extraction for MLS 1765-92, MLS 402-91, MLS 2645-94 and EWS TC-71.

Supplementary Table 3

Supplementary Table 3 (MS-identified SWI/SNF, Mediator and RNA polymerase II components after FET-FOP IP) is displayed in Supporting file 2 (.xlsx)
