# Supplementary material for: FET fusion oncoproteins interact with BRD4 and SWI/SNF chromatin remodelling complex subtypes in sarcoma
Source: Mol Oncol. 2022 Mar 19;16(13):2470–95. doi: 10.1002/1878-0261.13195 (PMC9251840; doi:10.1002/1878-0261.13195)
Supplement: Supplementary file 3 — Fig. S3. Quantitative western blot. [file MOL2-16-2470-s004.pdf]

Source Data WB for Figure 1 and Supplementary Figure 1

Fig. 1c and S1

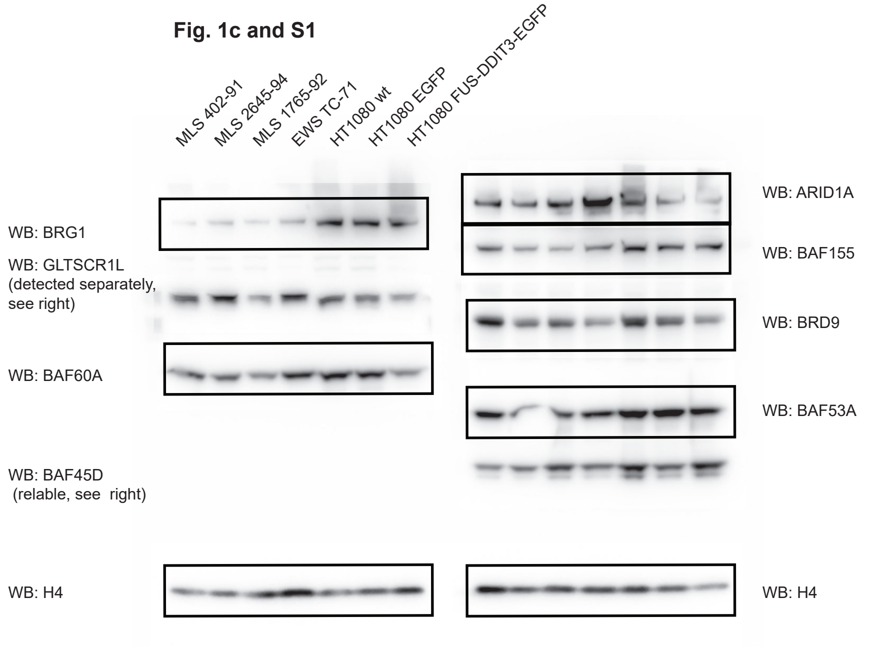

Fig. 1c and S1

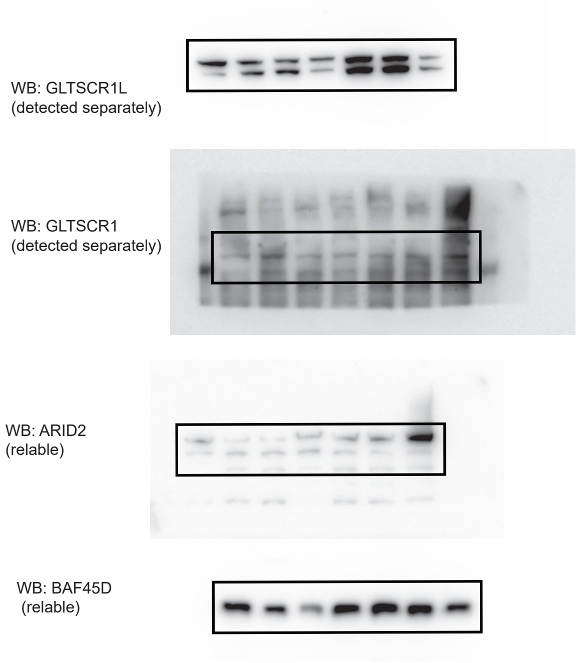

Fig. 1c and S1

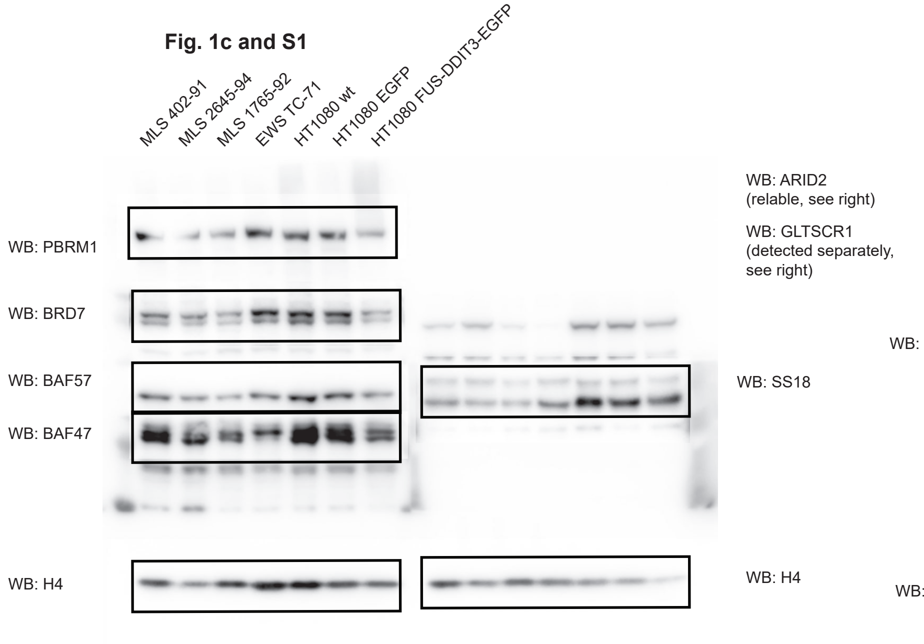

Fig. S1

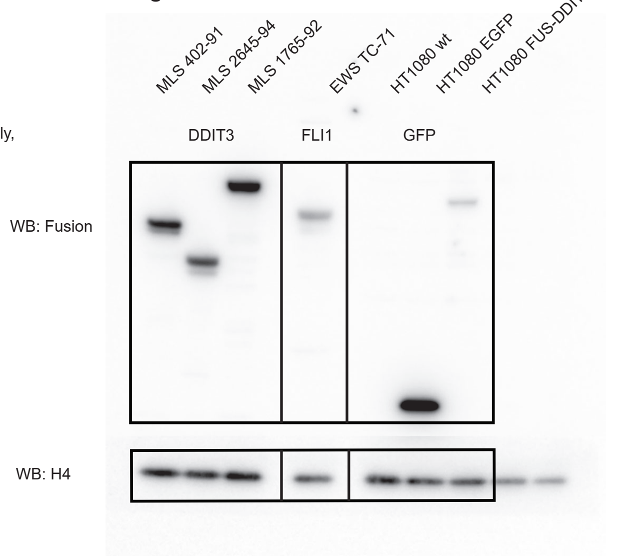

Fig. 1e

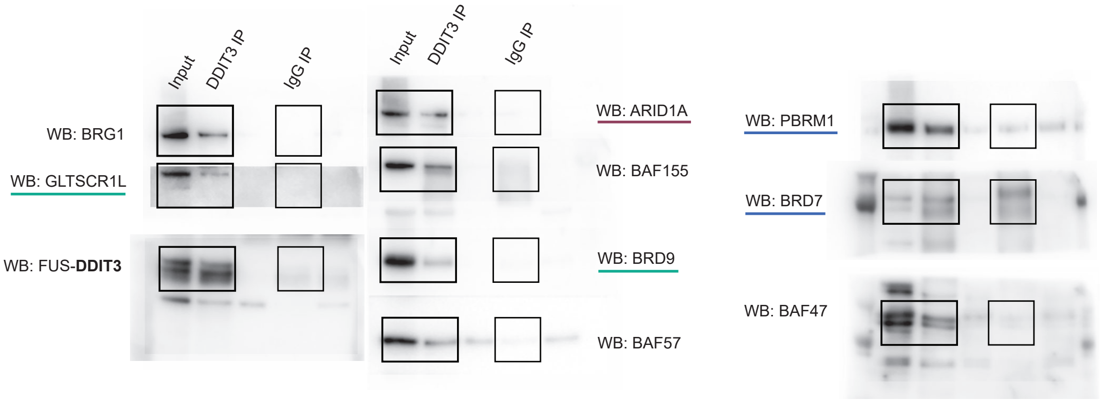

Source Data WB for Figure 2

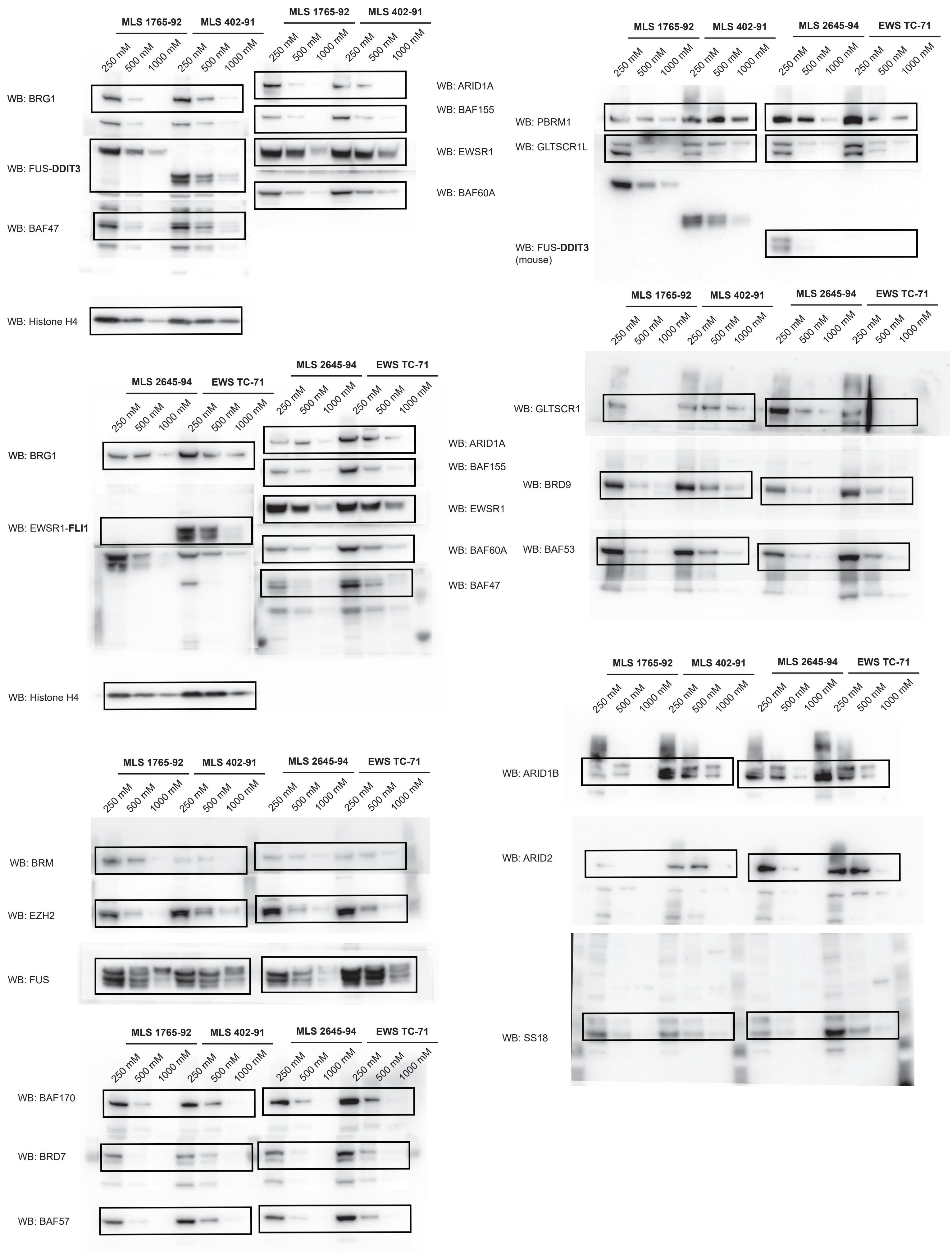

Source Data WB for Figure 3

Fig. 3a

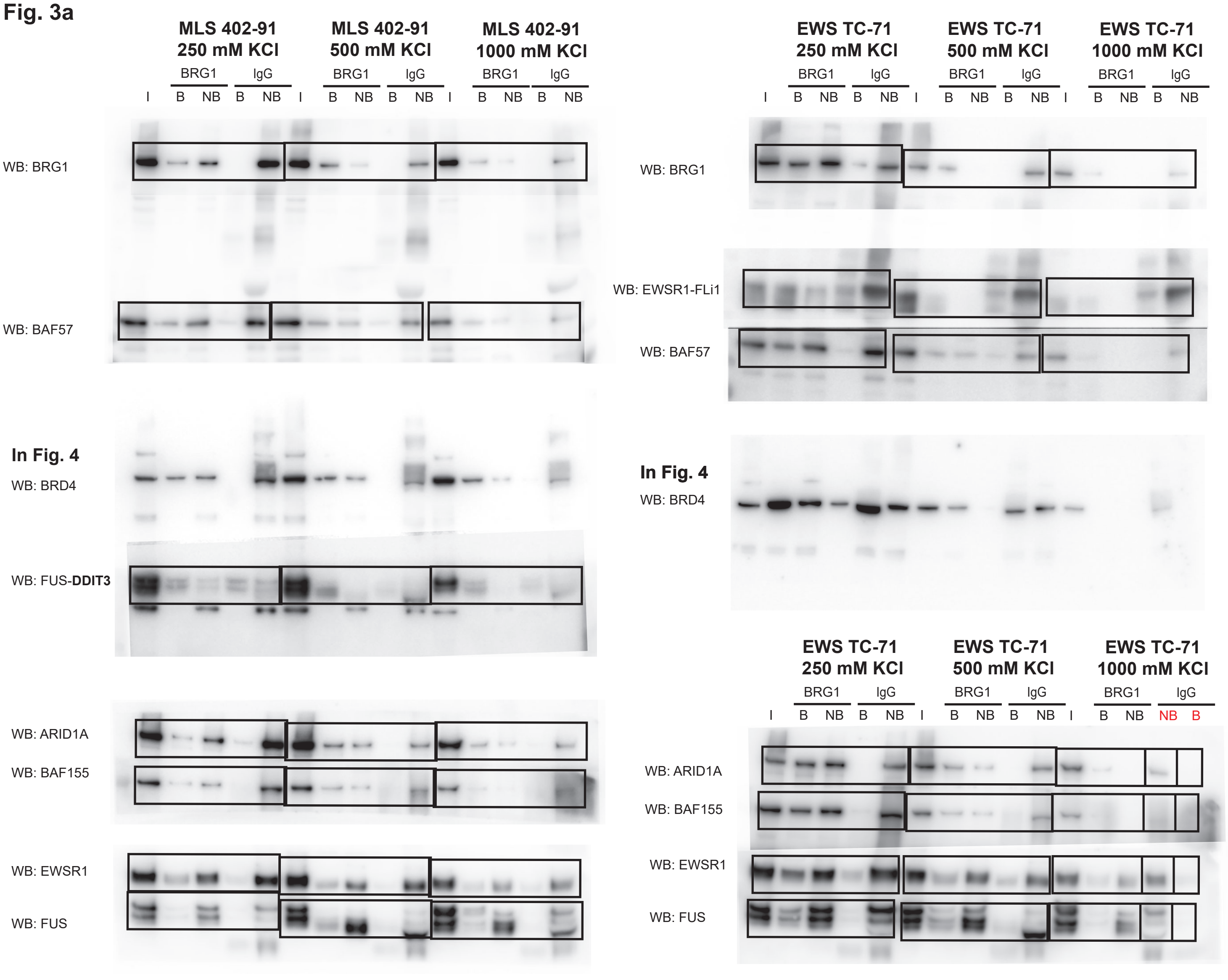

Fig. 3b

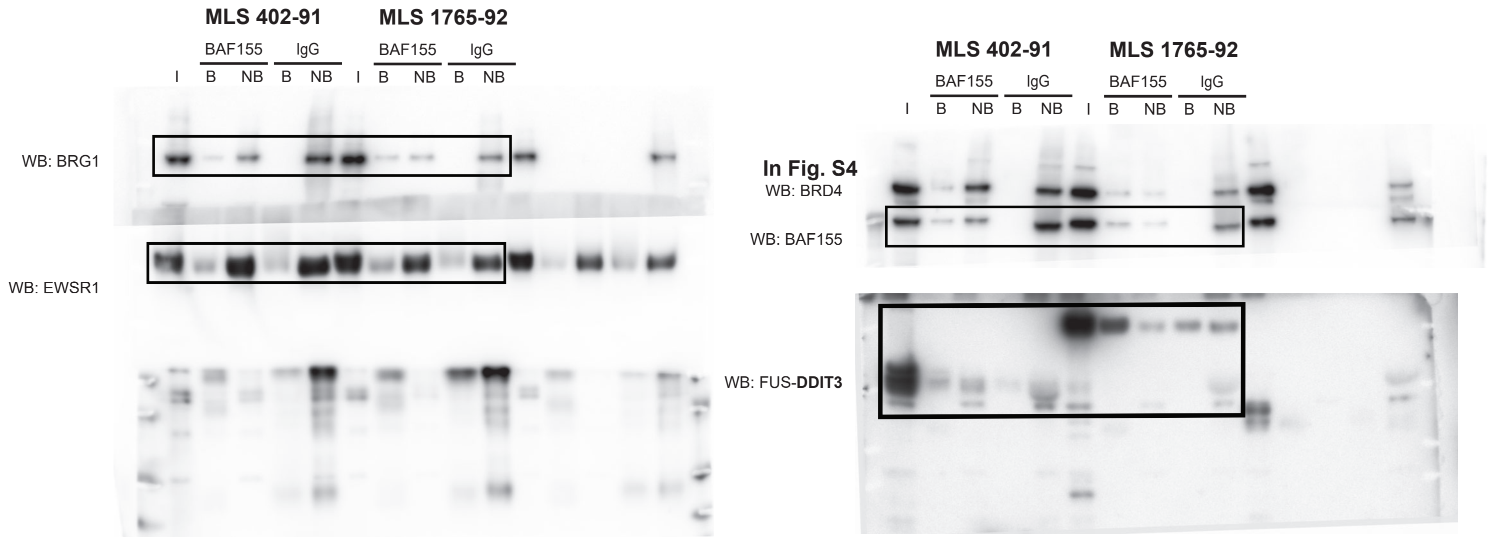

Source Data WB for Figure 4

Fig. 4b

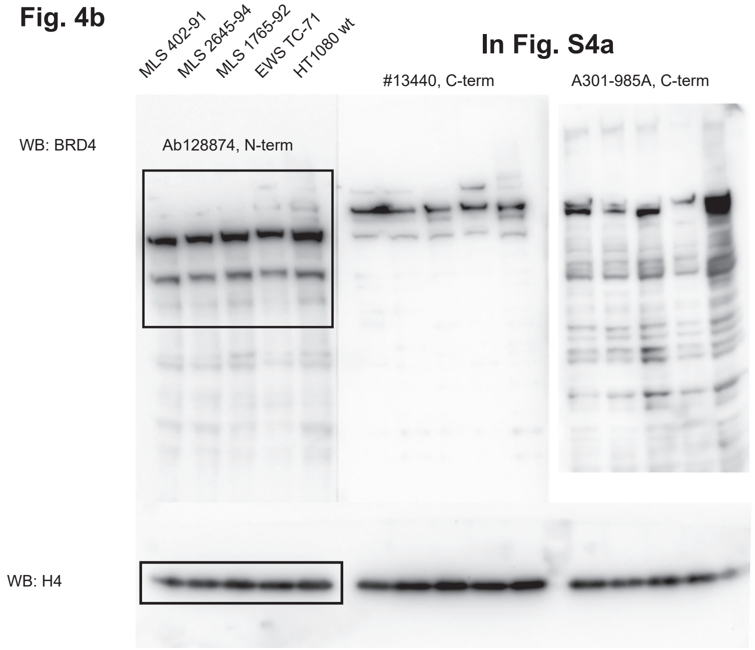

Fig. 4c

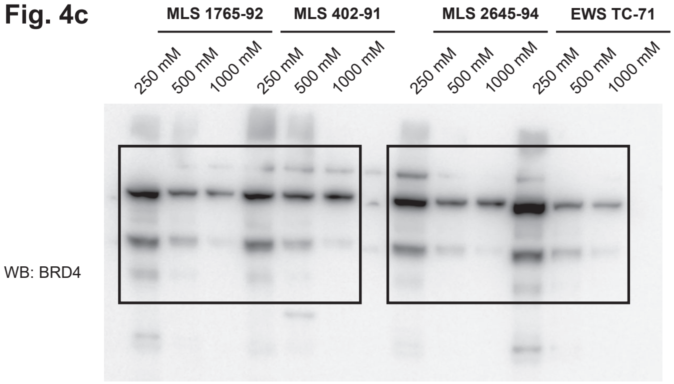

Fig. 4d

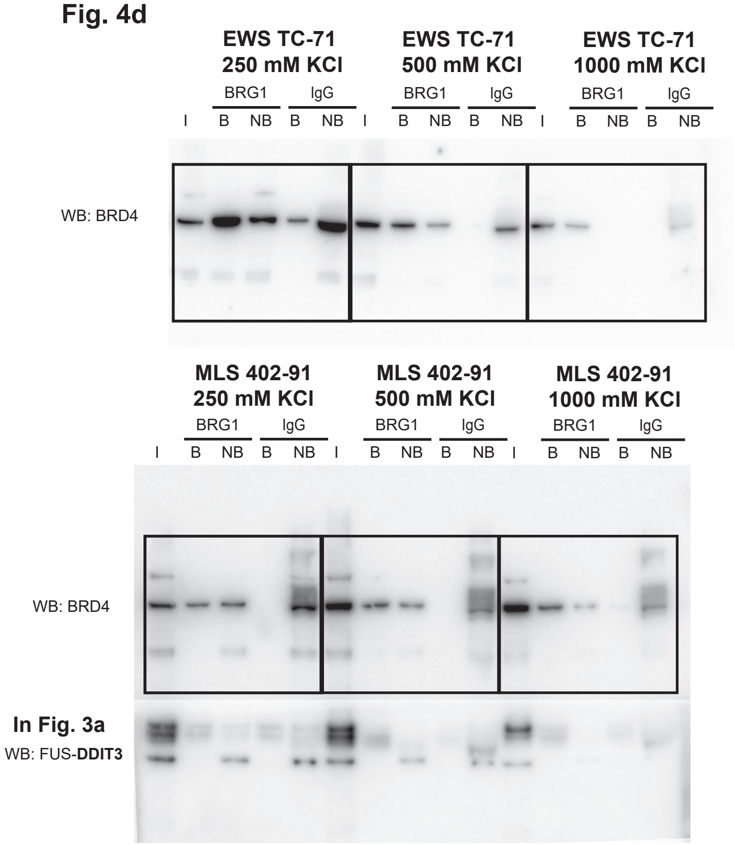

Source Data WB for Figure 4

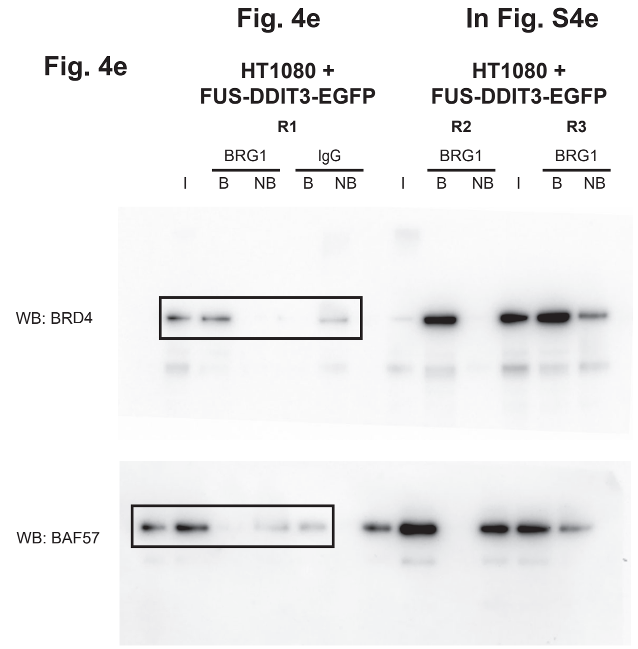

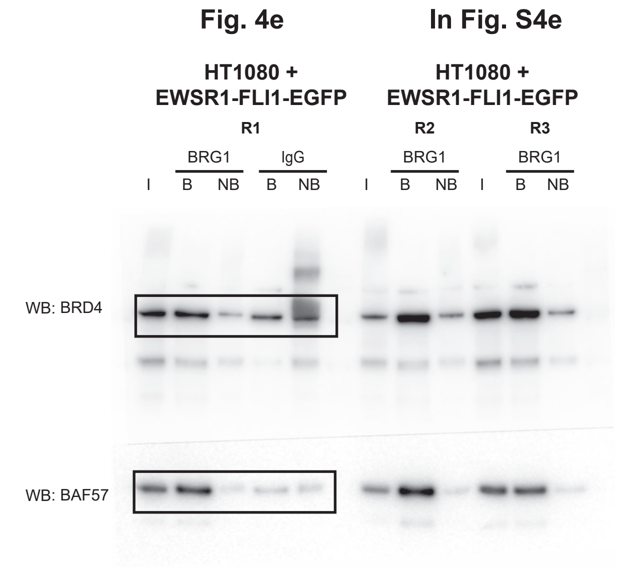

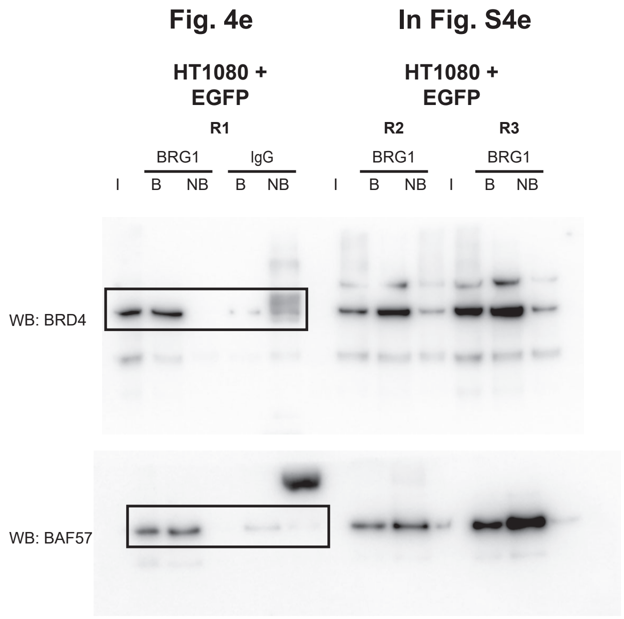

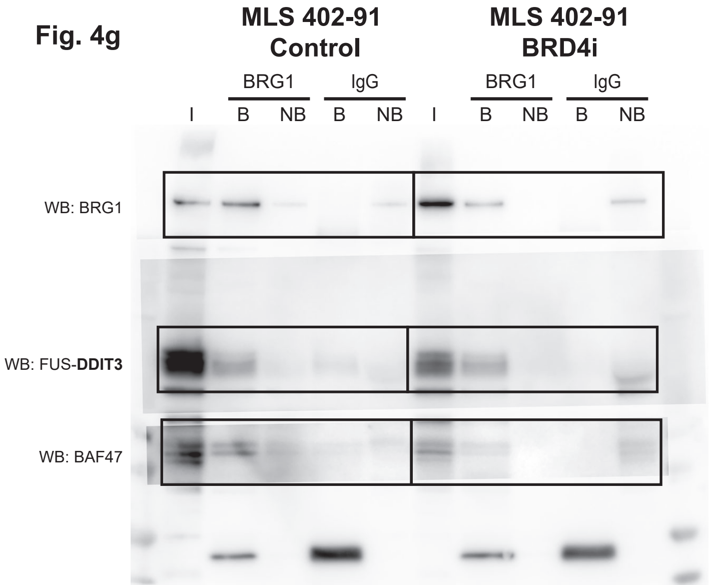

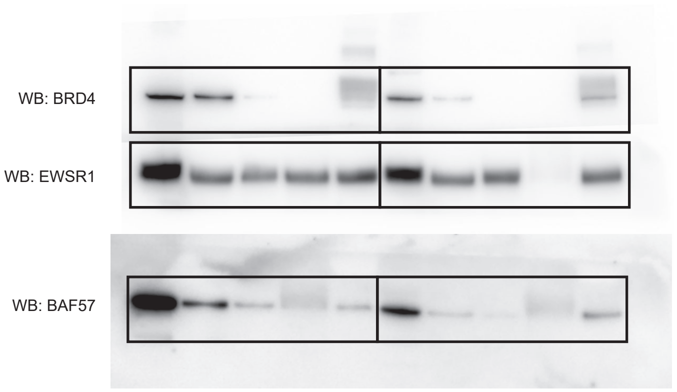

Source Data WB for Supplementary Figure 3

Fig. S3b

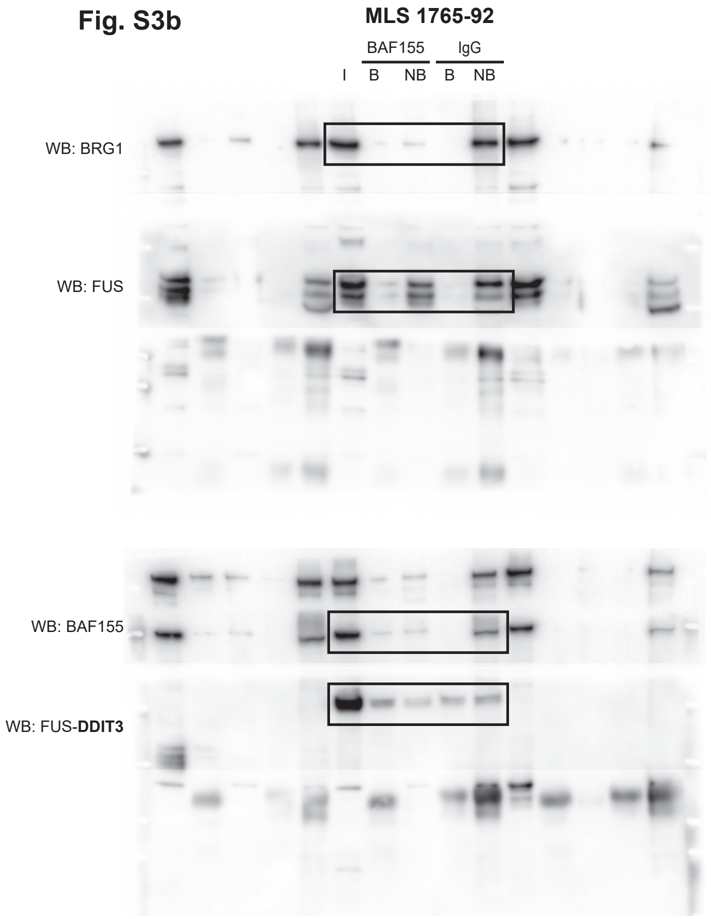

Source Data WB for Supplementary Figure 4

Fig. S4a

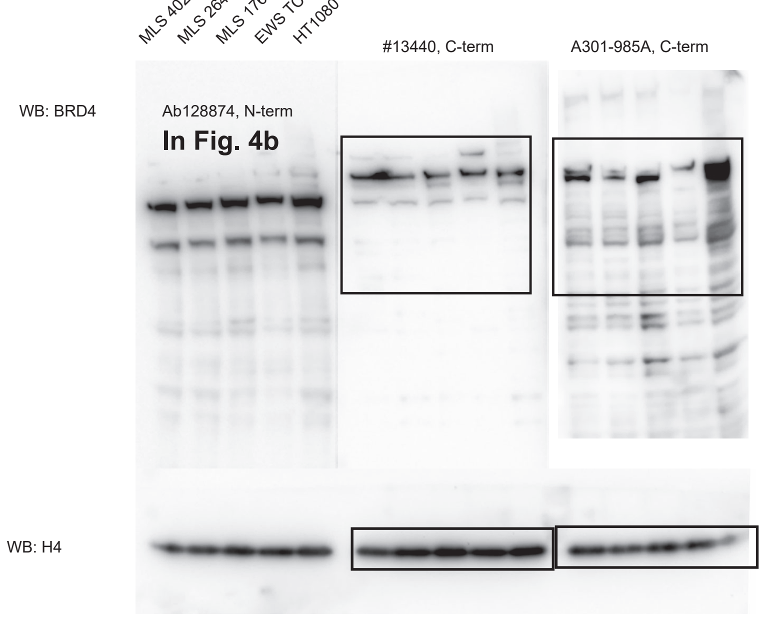

Fig. S4b

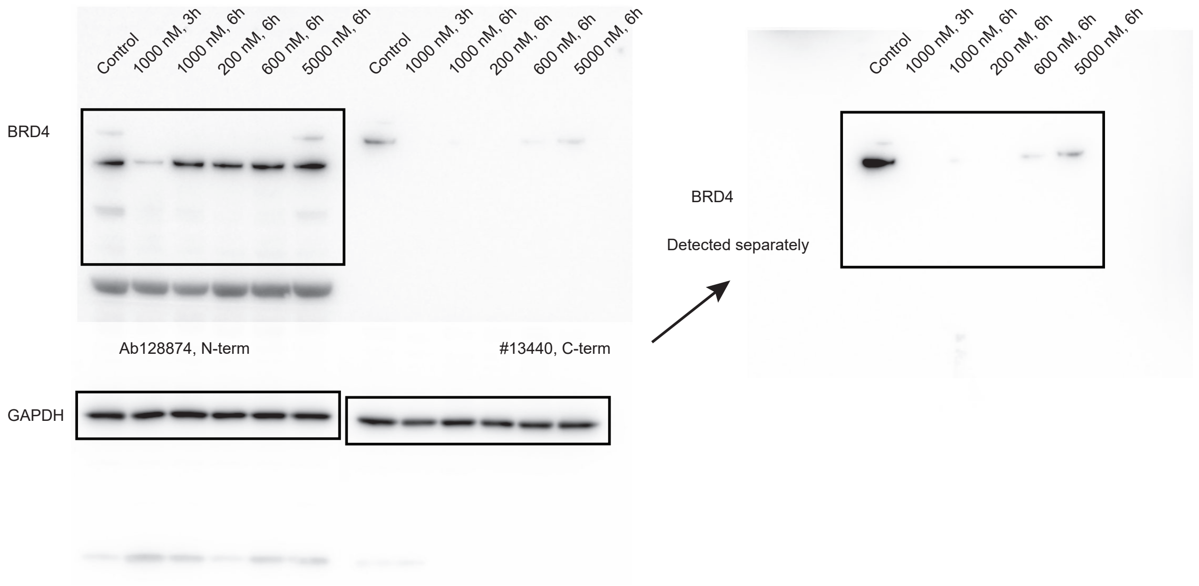

Fig. S4c

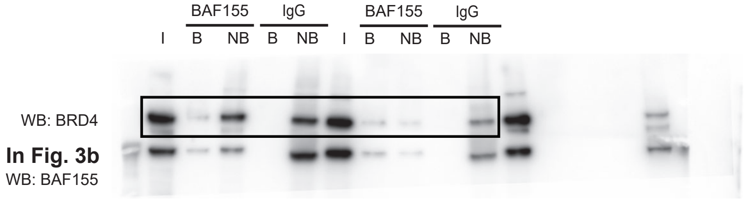

Fig. S4d

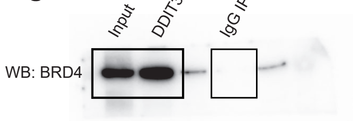

Fig. S4e

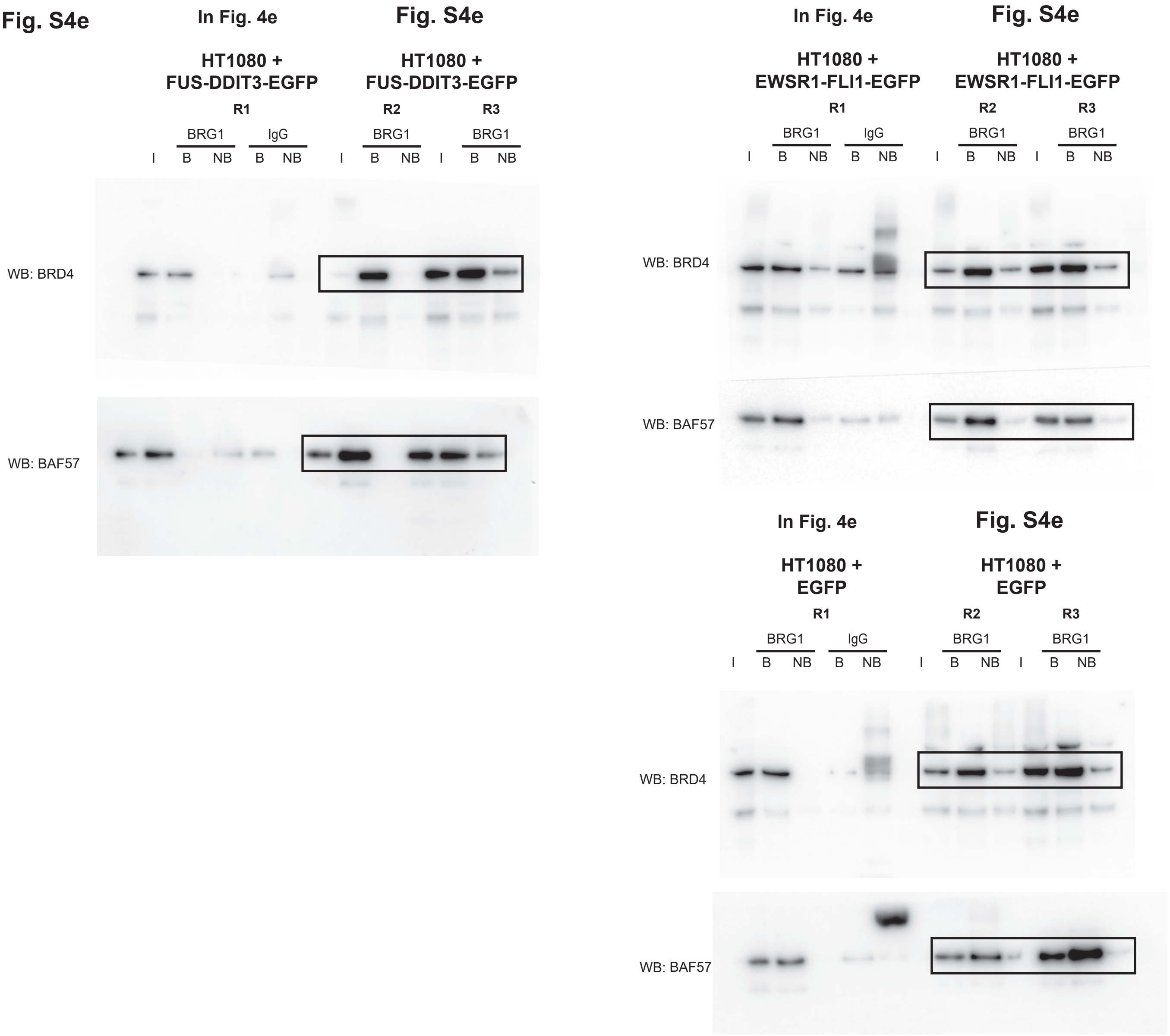
In Fig. 4e
Fig. S4e
HT1080 +
FUS-DDIT3-EGFP
HT1080 +
FUS-DDIT3-EGFP
R1
R2
R3
BRG1
IgG
BRG1
BRG1
I
B
NB
B
NB
I
B
NB
I
B
NB
WB: BRD4
WB: BAF57
In Fig. 4e
Fig. S4e
HT1080 +
EWSR1-FLI1-EGFP
HT1080 +
EWSR1-FLI1-EGFP
R1
R2
R3
BRG1
IgG
BRG1
BRG1
I
B
NB
B
NB
I
B
NB
I
B
NB
WB: BRD4
WB: BAF57
In Fig. 4e
Fig. S4e
HT1080 +
EGFP
HT1080 +
EGFP
R1
R2
R3
BRG1
IgG
BRG1
BRG1
I
B
NB
B
NB
I
B
NB
I
B
NB
WB: BRD4
WB: BAF57
